# Molecular Simulation-Based Structural Prediction of Protein Complexes in Mass Spectrometry: The Human Insulin Dimer

**DOI:** 10.1371/journal.pcbi.1003838

**Published:** 2014-09-11

**Authors:** Jinyu Li, Giulia Rossetti, Jens Dreyer, Simone Raugei, Emiliano Ippoliti, Bernhard Lüscher, Paolo Carloni

**Affiliations:** 1 Computational Biophysics, German Research School for Simulation Sciences (joint venture of RWTH Aachen University and Forschungszentrum Jülich, Germany), Jülich, Germany and Computational Biomedicine, Institute for Advanced Simulation IAS-5 and Institute of Neuroscience and Medicine INM-9, Forschungszentrum Jülich, Jülich, Germany; 2 Institute of Biochemistry and Molecular Biology, RWTH Aachen University, Aachen, Germany; 3 Jülich Supercomputing Centre (JSC), Forschungszentrum Jülich, Jülich, Germany; 4 Fundamental and Computational Sciences Directorate, Pacific Northwest National Laboratory, Richland, Washington, United States of America; University of Maryland, United States of America

## Abstract

Protein electrospray ionization (ESI) mass spectrometry (MS)-based techniques are widely used to provide insight into structural proteomics under the assumption that non-covalent protein complexes being transferred into the gas phase preserve basically the same intermolecular interactions as in solution. Here we investigate the applicability of this assumption by extending our previous structural prediction protocol for single proteins in ESI-MS to protein complexes. We apply our protocol to the human insulin dimer (hIns_2_) as a test case. Our calculations reproduce the main charge and the collision cross section (CCS) measured in ESI-MS experiments. Molecular dynamics simulations for 0.075 ms show that the complex maximizes intermolecular non-bonded interactions relative to the structure in water, without affecting the cross section. The overall gas-phase structure of hIns_2_ does exhibit differences with the one in aqueous solution, not inferable from a comparison with calculated CCS. Hence, care should be exerted when interpreting ESI-MS proteomics data based solely on NMR and/or X-ray structural information.

## Author Summary

Electrospray ionization (ESI) mass spectrometry (MS) plays a pivotal role in proteomics and structural biology. The applications of ESI-MS to protein complexes make use of the assumption that the vaporization of protein complexes into the gas phase (as occurs during ESI-MS) preserves the structural determinants of the complexes that are observed in water. We used computational methods to investigate this key issue by studying the gaseous structure of a pharmacologically relevant protein complex. The complex in the gas phase differs in a subtle yet significant way from the solution structure. This finding is likely of general relevance for protein-protein complexes. Hence, our work implies that the assumption used in proteomic studies, i.e. that in the gas phase non-covalent complexes generally preserve the representative structural determinants observed in the aqueous phase, needs to be reconsidered. Therefore we suggest that the analysis of complexes should be performed on an individual base rather than by generalized principles.

## Introduction

Proteomics, the large-scale characterization of proteins and their interactions, is key to understand cellular processes including signaling pathways, metabolism, and gene transcription [1]–[3]. Arguably, the most powerful tool for studying functional proteomics is protein electrospray ionization (ESI) mass spectrometry (MS) [3]–[11]. ESI-MS detects rapidly and reliably proteins in complexes formed during cellular processes at physiologically relevant concentrations [12]. It provides the stoichiometry, topology, connectivity, dynamics and shape of multi-protein complexes when combined with ion mobility (IM)-MS experiments [2], [13]. Using the IM-MS technique, collision cross sections (CCS) can be determined [2], [3], [13], [14] with protein concentrations well below those required for high resolution (X-ray and NMR) as well as low resolution traditional structural biology techniques [3], [6], [15], [16] such as electron microscopy [17] and tomography [18].

ESI-MS has also been used for structural proteomics in combination with experimental structural biology techniques (e.g. X-ray and NMR) and/or computational techniques (e.g. homology modeling and protein-protein docking) [3], [19]–[23]. These applications are based on the assumption that the vaporization of non-covalent protein complexes from aqueous solution into the gas phase (as occurs during ESI-MS) in general preserves the characteristic structural determinants of the complexes in water [24]–[28]. This assuption is consistent with the avaliable CCS data for some biomolecules and with the fact that intact non-covalent protein complexes in ESI-MS are indeed transferred into the gas phase [29]–[34]. However, direct proove for this concept has not been forthcoming at the atomistic structural level, because the structural determinants of gas-phase protein complexes have remained largely unknown [28]. Thus, the preservation of these determinants on passing from solution into the gas phase is still under debate for protein complexes. Predicting the structure of protein complexes under ESI-MS conditions, and in particular assessing whether native interactions in the gas phase reflect those in the aqueous phase, is therefore important for ESI-MS based structural proteomic studies.

A straightforward approach to improve the structural prediction is to run molecular dynamics (MD) simulations and select models that are consistent with the CCS [35], [36]. However, these investigations have limited predictive power as no validations are provided against the main charge and the simulation is basically used as a tool to generate structural ensembles from which specific conformers can be selected [35]. More elaborate protocols have been developed for single proteins in the gas phase [24], [37]–[47]. These approaches have predicted ensembles of structures consistent with the experimentally measured charge and CCS [38], [39]. They have further suggested that desolvation leads to more compact overall protein structures while preserving the majority of the secondary and tertiary structures [24], [37], [38], [40]. In addition, the fraction of hydrogen bonds (relative to the theoretical maximum) increases significantly upon passing from aqueous solution (on average 43%) to the gas phase (on average 56%) [41]. This suggests that proteins in the gas phase may be trapped in a low energy state, structurally close to the native state in water [42]. Our recent studies further indicate that the ionization state of a gas-phase protein is the result of the balance between repulsive electrostatic terms and stabilizing forces that include salt bridges, hydrogen bonds, π-charge and long-range electrostatic interactions [37], [38]. Therefore, these simulation schemes appear instrumental to predict the structural determinants of protein complexes.

Recently, we have proposed an efficient approach to sample exhaustively the proteins' protonation state space, based on a hybrid Monte Carlo (MC)/MD scheme [38]. Here, we extend this computational scheme, originally developed for single protein ESI-MS structural predictions, to a protein complex, the human insulin dimer (hIns_2_ hereafter, supporting information (SI), Figure S1). hIns_2_ is present *in vivo* [48]. It is used for the treatment of diabetes and obesity [49], [50]. Our predictions reproduce the experimental main charge state and CCS. They further show that, in the sub-ms time scale (possible times of the ESI-MS experiments to form stable gas-phase structures, ranging from ms to s [28]) the overall gas-phase structure of the complex rearranges already significantly. The final gas-phase structure differs distinctively from the solution structure as large amplitude reorganizations take place in order to maximize intra- and intermolecular hydrogen bond interactions, which are necessary for the formation of stable gas-phase structures. Hence, our current work provides evidence against the assumption that non-covalent complexes being transferred into the gas phase generally preserve their structural determinants in solution.

## Results/Discussion

Here (i) we first employed our hybrid MC/MD scheme-based protocol, used for single proteins [38], to explore the protonation state space of hIns_2_ and to identify the main charge state and its most probable conformer. Then, (ii) we performed sub-ms MD simulations on the latter in the gas phase to investigate its structural features. Both steps were validated by comparison with experiments. Comparison with independent MD simulations of the protein dimer, with different initial condition and/or force field was additionally made.

(i) The protocol developed by us and used here for protonation state space exploration [38] was applied to hIns_2_ in the gas phase with different charge states ([hIns_2_]^q^, q = 1+,2+,…,15+) (see the Methods and Text S1). The protocol uses both MC and MD simulations and is based on standard force field energy augmented by additional energy terms associated with the gas-phase basicity (GB) of ionizable residues [38]. The initial structure of our calculations was taken from MD simulations of the protein complex in water at physiological pH, which was in turn based on a high resolution X-ray structure (see SI, Figure S1 for further details).

**Figure 1:**
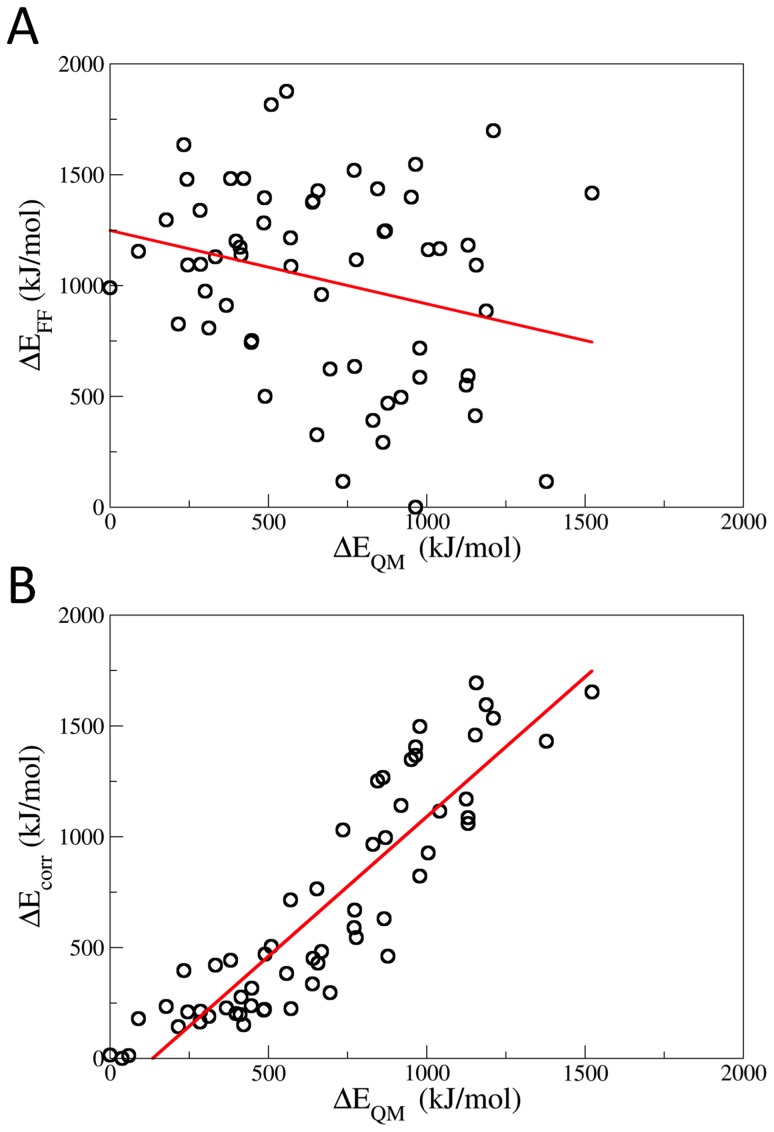
Correlation plots of the differences in energy for 60 protonation states of the hIns_2_ relative to the lowest-energy protonation state. (A) Energy differences calculated with the OPLS/AA force field (ΔE_FF_) [63] versus differences calculated with DFT (ΔE_QM_) [38]. (B) Energy differences calculated with the OPLS/AA force field along with the GB correction (ΔE_corr_) versus differences calculated with DFT (ΔE_QM_). The correlation is much better with GB correction than with OPLS/AA (*R* ^2^ = 0.81 and 0.03, respectively), confirming the crucial role of GB for estimating the energies of the protonation states.

**Figure 2:**
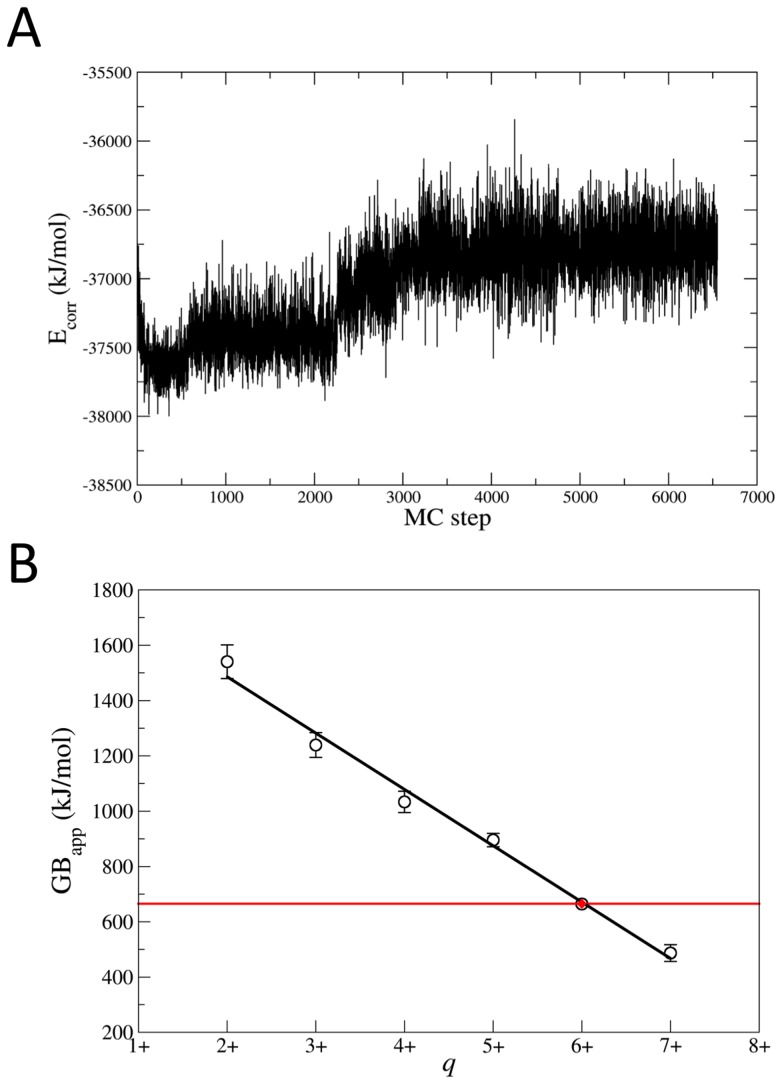
Protonation state space exploration. (A) GB corrected energy (E_corr_) as a function of MC step for the MC/MD sampling on hIns_2_ at the main charge state (q = 6+). (B) Prediction of the main charge state of hIns_2_. GB_app_ values (in kJ/mol) were calculated for the lowest energy protonation states of hIns_2_ (black line and cycle symbols). Standard deviation from the average is given as error bars. When not visible, the standard deviation is smaller than the symbol size. The red horizontal line indicates the GB of water (660.3 kJ/mol taken from ref. [101]). The experimental main charge state [52] is shown by red solid diamond.

The GB corrected energies correlate with density functional theory (DFT) results much better than the energies derived from the original force field (Figure 1, see the Methods for further details). The corrected energies of the complex for fifteen charge states turned out to decrease largely already after few hundred steps (Figure 2A for the case of [hIns_2_]^6+^ and SI, Figure S2 and Figure S3). The identified lowest energy protonation states for each charge state are reported in Table S1.

The protocol was validated by predicting the experimental main charge (which is usually the maximum charge for folded proteins [51]) of the complex under ESI. This is q = 6+ [52]. We used the fact that the charge state of protein ions with the apparent gas-phase basicity (GB_app_, see its definition in the Text S1) close to the GB of the solvent, from which the protein ions are formed, reproduces the experimental maximum charge states under ESI. The theoretical values are within 6% of the experimental values for 13 proteins [53]. Following published procedures [53], [54], we estimated the maximum charge by calculating the intersection of the GB_app_ fitted line as a function of protein complex net charge with the line of the solvent GB. The intersection occurs at q = 6+ (Figure 2B), matching the experimentally measured main charge for hIns_2_ generated from a solution at pH = 7.4 [52].

(ii) 0.075 ms long MD simulations in the gas phase were performed on the lowest energy protonation state for the main charge state, i.e. [hIns_2_]^6+^. The simulations appeared to be gradually equilibrated already after ∼55 µs as indicated by the convergence of the backbone heavy atoms root mean square deviations (RMSD) of the complex and other structural properties (e.g. radius of gyration (*R*_g_) and center-of-mass distance between monomers) as a function of simulated time (Figure S4A to Figure S4D). The convergence of the simulations has been probed by the cosine content of the first principal component (PC) according to the Hess method [55]. When the cosine content is close to 1, it means that the system is far from convergence. The cosine contents of our systems turn out to be close to 0, indicating a good sampling of insulin dimer conformations (see Text S1 for details).

**Figure 3:**
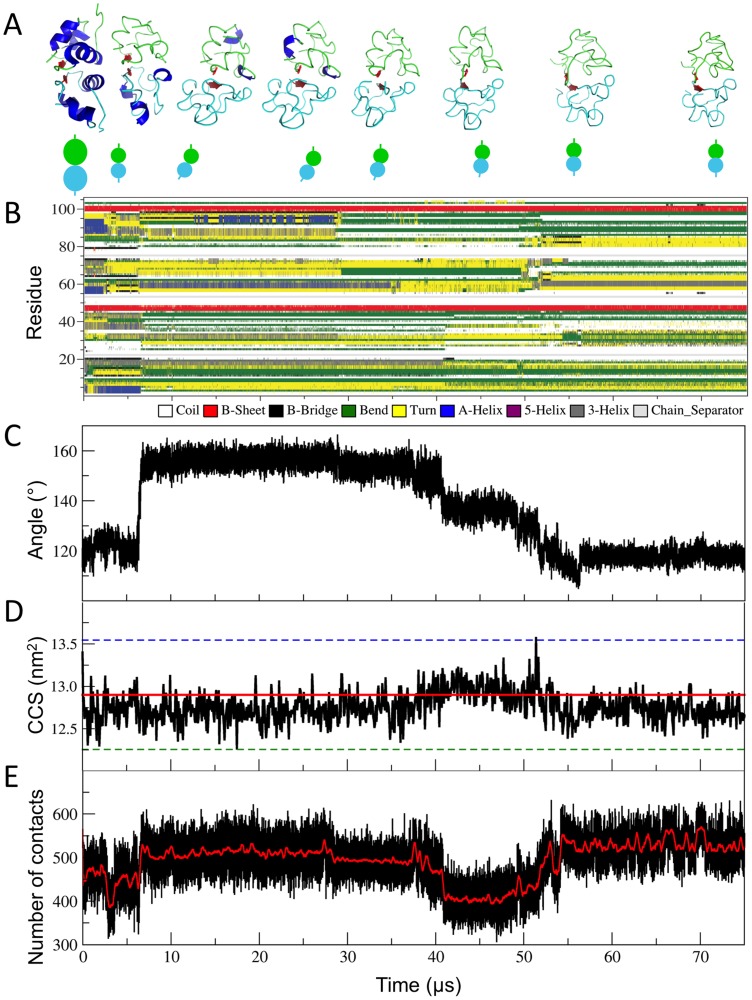
MD simulations in the gas phase of the [hIns_2_]^6+^. (A) Models of [hIns_2_]^6+^ obtained from MD simulations in the gas phase (from left to right, at 0 µs, 5.7 µs, 8.1 µs, 27.6 µs, 36.3 µs, 42.6 µs, 54.9 µs, and 75.0 µs). The monomer I and II are indicated in cyan (lower structure) and green (upper structure), respectively. The α-helices and β-sheets are highlighted in blue and red, respectively. Schematic representations of the complex models are shown below the complex structures, at corresponding positions on the simulation time axis. The backbone RMSD values of the models in respect to the one at 0 µs are 0.25 nm (5.7 µs), 0.54 nm (8.1 µs), 0.55 nm (27.6 µs), 0.49 nm (36.3 µs), 0.45 nm (42.6 µs), and 0.49 nm (54.9 µs), and 0.50 (75.0 µs). (B) Secondary structure analysis for [hIns_2_]^6+^. (C) The angle between the center of mass (COM) of monomer I – β-sheet region – monomer II. (D) CCS values. The experimental value of 12.9 nm^2^, as reported [52], at the main charge state is indicated by a red solid line and its 5% variations are indicated by the dashed lines. The average value from our MD simulation in the gas phase is 12.8±0.2 nm^2^. (E) Number of contact pairs between the carbon atoms of the monomers within 0.60 nm.

Overall our simulations indicate that the β-strand secondary elements are more stable than the α-helices, i.e. the average contents of β-sheets in water and in the gas phase are 4.0±1.2% and 3.9±1.1%, respectively, while the ones of α-helices are 38.7±3.0% and 2.8±4.0%, respectively. These findings are consistent with the lower stability of α-helices than β-sheets in the gas phase observed from previous simulations [56], [57]. Specifically, the two-stranded antiparallel β-sheet motif at the interface of the two insulin monomers was well maintained during MD simulations (Figure 3A and Figure 3B). This motif was stabilized by monomer-monomer hydrogen bonds in solution (Figure S1E), such as in [58], [59]. In contrast, the six α-helices, stabilized by hydrophobic interactions in solution (Figure S1F), unfolded after ∼25 µs (Figure 3A and Figure 3B).

**Figure 4:**
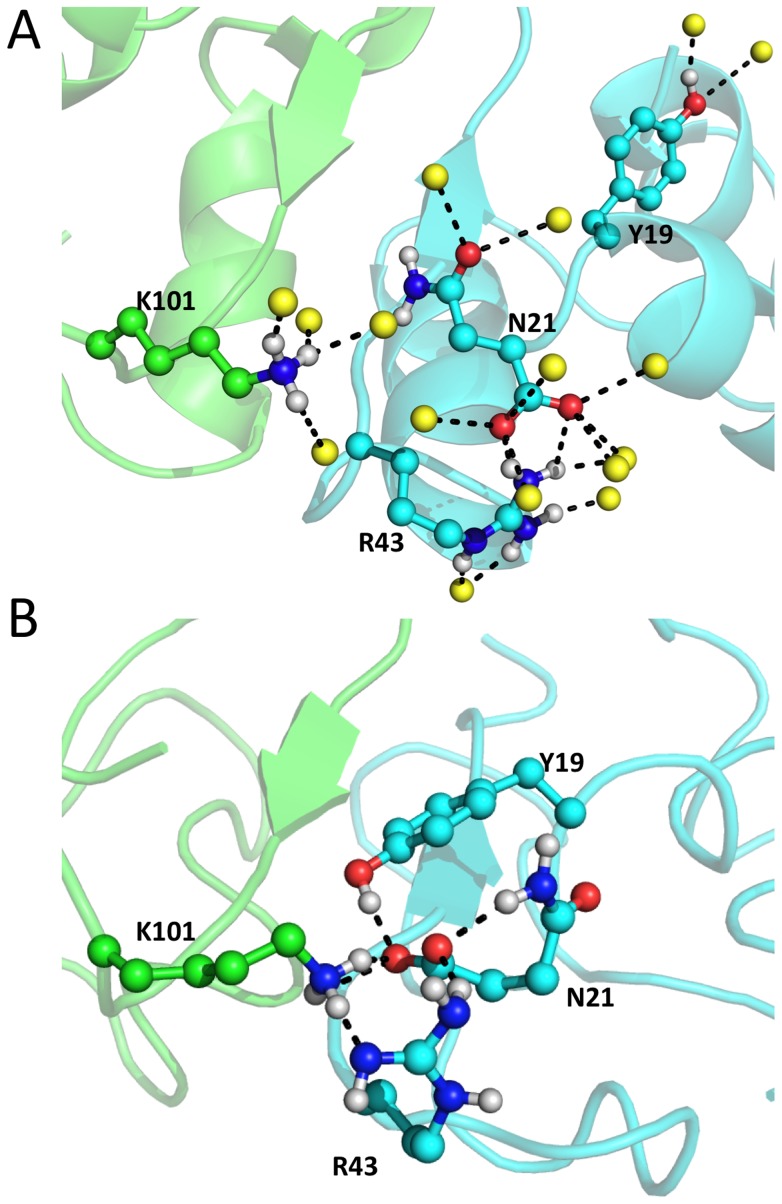
Comparison of a local inter- and intra-molecular hydrogen bond network in water (A) and in the gas phase (B). The final snapshots obtained from the MD simulations in water and in the gas phase at the main charge were selected. The monomer I and II are indicated in cyan and green, respectively. The water oxygen atoms are indicated by yellow balls. Nitrogen, dark blue; oxygen, red; hydrogen, white. Hydrogen bonds are shown as dashed lines.

Our MD simulations of the protein complex in solution suggest that 230.2±8.6 hydrogen bonds are formed between the protein complex and the solvent. A significant fraction of these (∼12%) is replaced by hydrogen bonds within the protein complex on passing from solution into the gas phase. Hence, several of the protein hydrogen bonds functionalities, forming hydrogen bonds with the solvent, rearrange in the gas phase so as to form intra- and intermolecular hydrogen bonds not present in solution. Figure 4 shows the reorganization of one of the hydrogen bond networks between solution and gas phase (panel A and B, respectively). In contrast, the intermolecular van der Waals contacts did not reveal significant changes (Table S2). This may be due, at least in part, to the fact that these contacts are maximized both in solution (because of the hydrophobic effect [60]) and in the gas phase.

The *R*_g_ of the complex in the gas phase (1.30±0.01 nm) decreased compared to the one in water (1.37±0.01 nm, see Figure S4A and Table S2). This is consistent with previous simulations both on single proteins and protein complexes [35], [37], [38].

During the simulations we also observed progressive rearrangements of the two insulin monomers with respect to each other. Specifically one of the two monomers (monomer I (cyan) in Figure 3A) first rotated by about 30 degrees relative to the other after 6 µs (Figure 3C), with additional small rearrangements when the helices unfolded (Figure 3B), and then stepwise rotated backward by about 20 degrees between 28 µs and 55 µs. The angle values between monomer I – β-sheet – monomer II at the end of the MD simulation were similar (∼1.5 degree of difference) to the initial values.

Accordingly, the evolution in time of the number of hydrogen bonds within the whole complex and those between monomers decreased (from 90.3±4.6 to 80.6±4.7 and from 14.6±1.6 to 13.4±1.5, respectively) after 6 µs and stepwise increased (from 80.6±4.7 to 92.8±4.6 and from 13.4±1.5 to 15.3±1.5, respectively) from 28 µs to 55 µs (Figure S4E and Figure S4F). At the end of the simulation, these numbers were larger than those of the complex in water (Table S2). Stepwise rearrangements to maximize inter- and intra-molecular hydrogen bonds in the formation of gas phase structures has also been observed in monomeric proteins [61]. In contrast, the number of van der Waals contacts first increased and then decreased, then increased again and finally were maintained in the latter part (after 55 µs) of the simulations (Figure 3E). At the end of the simulation the number was comparable to the starting situation, as we discussed above.

Next, we investigated the largest scale motions of the system by essential dynamics analysis (EDA) [62] (see Methods). In the combined water-and-gas-phase trajectories (see Methods for details), the largest scale motion involves a fast compaction of the complex and an unfolding/refolding transition of the α-helices (Figure S5A). Instead in the converged part of trajectory of the gas phase (i.e. the latter 0.020 ms, see discussion above and Figure 3) the largest scale motion entails a twisting of each subunit relatively to the other (Figure S5B). This suggests that after the system achieves equilibration in the gas phase, the compaction motion is less relevant than in solution, possibly because of the observed structural changes on passing from water into the gas phase. Notably, the largest scale motion calculated for the entire simulation in the gas phase is similar to that of the combined water-and-gas-phase trajectories (Figure S5C). Thus, the initial, non-equilibrated gas phase motion retains a “memory” of the simulation in solution.

Importantly, the calculated CCS values obtained from the gas phase simulations (12.8±0.2 nm^2^), reproduced the experimentally determined value (12.9 nm^2^) [52]. However, we found that the calculated CCS values were not sensitive enough to detect the subtle, yet significant structural arrangements described above (Figure 3 and Table S3). Indeed, the calculated CCS shows no correlation with other gas-phase structural properties (Figure S6). As a further test to prove this issue we also calculated the CCS (Table S3) before (e.g. at time 5.7 µs) and after (e.g. at time 8.1 µs) the turn of monomer I. The CCS variation is about 0.1 nm^2^ (from 12.6 nm^2^ to 12.7 nm^2^), a value within one standard deviation from the average values.

Finally, to check the dependence of conformational dynamics of the complex on the microscopic initial conditions and on the force field, we performed additional MD simulations (see Table S2 and Figures S7, S8 and S9) on the lowest energy protonation state for the main charge state (q = 6+) in the gas phase. Specifically, we performed (1) two additional 0.035 ms long OPLS/AA-based [63] vacuum independent simulations with different starting velocities and (2) one additional 0.025 ms long vacuum simulation with the GROMOS 43a1 force field [64]. Selected averaged structural properties calculated from these simulations are similar to each other (Table S2). The only exception is the slightly more compact structure obtained from the GROMOS 43a1-based simulation. This may be due, at least in part, to the overestimation of London forces in this force field [65]. Taken altogether, these results indicate that our calculations are basically independent of the initial microscopic conditions and the adopted force field. Despite the similarities of the observed average structural properties, several possible pathways and intermediate conformers exist upon transfer from water into the gas phase (Figures 3, S7, S8 and S9), consistently with what has been observed previously [66]–[68].

### Conclusions

We have reported a systematic exploration of the charge and conformational space of the hIns_2_ non-covalent complex in the gas phase by using a hybrid MC/MD approach and sub-millisecond MD simulations. The long time required for observing structural changes such the unfolding of the helices (∼25 µs), as well as other conformational rearrangements, confirms that conformational changes in the gas phase may happen over long time scales (from µs to ms) [28], [69], [70]. Our calculations correctly reproduce the experimental main charge and the CCS measured in solution at pH = 7.4 [52]. Hence, molecular simulations approaches such as the one reported here may be a useful tool to (study and) complement the structural analysis of protein complexes via ESI-MS. We suggest that distinct protein complexes differ from one another when their structural properties are determined in gas phase or in solution. This is due to a substantial structural reorganization as a consequence of the maximization of intra- and intermolecular hydrogen bond interactions, which are necessary for the formation of stable vacuum structures.

Therefore, care should be exerted when interpreting ESI/IM-MS data that are solely based on NMR and/or X-ray structural information. Consistent with this, recent experimental work also illustrates that the comparison between measured and calculated CCS based on X-ray structures can only provide a semi-quantitative estimate [71]–[74]. This may be attributed to the considerable uncertainties (from 0 to ∼40%) involved in the experimental measurements of CCS related to drag enhancement of protein ions in the drift tube and other factors [71]–[73], as well as to the compaction of protein structure in the gas phase in comparison to the corresponding X-ray crystal structure [73].

Computational approaches such as ours or those by other groups [24], [56], [75], may therefore be instrumental to understand how desolvation affects the structure and stability of other protein complexes. Such simulations may establish whether the present findings can be generalized. This type of calculations may be of help for the development of efficient strategies to optimize experimental factors to control the gaseous protein ion structure in ESI-MS experiments.

## Methods

We first performed MD simulations in water based on the X-ray structure of hIns_2_ (1.0 Å) (PDB ID: 1MSO [76]). The protonation states of residues in solution were assigned according to the corresponding p*K*_a_ values calculated by using the H++ webserver [77]. As a result, H26, H31, R43, K50 and N-terminal residues (G1, F22, G52 and F73) were positively charged and E4, E17, E34, E42 and C-terminal residues (N21, T51, N72 and T102) were negatively charged. The total charge of the complex is 0. hIns_2_ was inserted into a water box with edges of 71×52×63 Å^3^ (in total 22,519 atoms). The AMBER ff99SB-ILDN force field [78]–[81] and TIP3P force field [82] were used for the protein complex and for water, respectively. Periodic boundary conditions were applied. Electrostatic interactions were calculated using the Particle Mesh Ewald (PME) method [83], and the cutoff for the real part of the PME and for the van der Waals interactions was set to 0.9 nm. All bond lengths were constrained using the LINCS algorithm [84]. Constant temperature and pressure conditions were achieved by coupling the systems with a Nosé-Hoover thermostat [85], [86] and an Andersen-Parrinello-Rahman barostat [87]. A time-step of 2 fs was employed. The protein complex underwent 1000 steps of steepest-descent energy minimization with 1000 kJ·mol^−1^·Å^−2^ harmonic position restraints on the protein complex, followed by 2500 steps of steepest-descent and 2500 steps of conjugate-gradient minimization without restraints. The system was then gradually heated from 0 K up to 300 K in 20 steps of 2 ns. 100 ns long MD simulation at 300 K and 1 atm pressure was carried out using GROMACS 4.5.5 [88]. The structure nearest to the average conformation of the complex in aqueous MD simulation (see Figure S1) was employed as starting structure for the MC/MD exploration of the protonation state space. The solvent molecules were removed.

The MC/MD simulations (see Text S1 for details) were based on the OPLS/AA [63] force field energies augmented by additional energy terms associated with the GB of ionizable residues [38]. To validate the augmented term, the energies of 60 selected protonation states for q = 6+ without and with the GB correction, as well as with DFT were calculated using the Becke exchange and Lee-Yang-Parr correlation functional (BLYP) [89], [90] and the TZV2P Gaussian basis set [91]. As in ref. [37], [38], [92], only the N-terminal, C-terminal, R, K, H, Q, D, and E residues were allowed to protonate or deprotonate. We chose the OPLS/AA [63] force field because it offers the most complete set of base/conjugate acid pairs for these residues, e.g. the force-field parameters for the deprotonated arginine residue are missing in AMBER [93] or CHARMM [94] force fields. Issues related to a particular choice for the force field have been carefully addressed in our earlier work [37], [38]. Specially, we showed that three different force fields (GROMOS 41a1 [64], AMBER99 [93], and OPLS/AA [63]) give the same gas-phase charge state for nine proteins of different size and fold, when the calculations were limited to protonation states containing the ionized residues common to all of the three force fields [38]. We considered protonation states at total charge states from q = 1+ to q = 15+ (this includes the experimentally measured q = 6+ [52]). The MC/MD protocol converged after a number of MC steps in the range of 1,500 to 6,500, depending on the charge state (over a total of ∼4,000 to ∼120,000,000 possible protonation states for each charge, see Table S4) were performed for various charge states.

The lowest energy protonation state for the main charge state (q = 6+) underwent MD simulations at 300 K for 0.075 ms in the gas phase with the same setup as the one described for the aqueous MD simulation, except that the time-step was 1.5 fs and the force fields was OPLS/AA [63]. To check for dependence on the microscopic initial conditions, additional two MD simulations, each 0.035 ms long, on the same protonation state were performed using different starting velocities. To check for the dependence of the results from the force field, we also performed 0.025 ms long MD simulation using GROMOS 43a1 [64]. The latter force field along with OPLS/AA [63], unlike others such as AMBER [93] and CHARMM [94], have standard parameters for deprotonated arginine residues. The latter are present in the identified lowest energy protonation state of [hIns_2_]^6+^ (see Table S1). Furthermore, MD simulations on other lower energy protonation states at the main charge state, with charges located on different residues, have been also carried out (see Table S5, Table S6 and Text S1).

Secondary structure elements were detected by using Define Secondary Structure of Proteins (DSSP) [95]. All figures for the visualization of structures were drawn using PyMOL (Molecular Graphics System, Version 1.3, Schrödinger LLC). CCS values were calculated for structures every 73.5 ns using the trajectory method [96] implemented in the MOBCAL code [97]. The EDA [62] was carried out for the whole (0.010 µs long) trajectory in water combined with the whole (0.075 ms long) trajectory in the gas phase, for the whole gas-phase one alone and for the converged part (0.055 to 0.075 ms) of the trajectory in the gas phase. The EDA was performed after iterative superposition of the MD trajectories on the crystal structure of hIns_2_. The ProDy (Protein Dynamics & Sequence Analysis) interface [98] implemented in VMD1.9.1 [99] was used for the visualization of EDA. The MC calculations were carried out using standard Metropolis sampling [100] written as a bash/awk shell script, the MD using GROMACS 4.5.5 [88].

## Supporting Information

Figure S1 **MD simulation of hIns_2_ in water.** (A) Primary sequence of hIns_2_ (each monomer consists of two chains of 21 and 30 amino acids linked by 2 disulfide bridges derived from a precursor molecule). The letters colored in red and blue represent chargeable sites of acidic (E, D, and C-terminal) and basic groups (R, K, H, and N-terminal), respectively, in solution. (B) hIns_2_ X-ray structure (PDB ID: 1MSO [76]). Monomer I (residues 1–51) and II (residues 52–102) are colored in blue and red, respectively. Each insulin monomer is composed of two peptide chains (A and B, colored in dark and light, respectively) linked by two disulfide bonds (shown as green sticks, sulfur atom in yellow). (C) Backbone atoms RMSD (in nm) from the starting conformation of hIns_2_ during the 100 ns long MD simulation in water. RMSD of the entire hIns_2_, of monomer I, and of monomer II are colored in black, red, and green, respectively. (D) B-factor (in Å^2^) plotted for Cα atoms of hIns_2_ from MD simulation and X-ray. The last 5 ns long MD trajectory of hIns_2_ has been used in the calculation of B-factors. The experimental values are obtained from the X-ray structure data of hIns_2_ [76]. Residues of chain AI and BI in monomer I are numbered 1–21 and 22–51, respectively. Residues of chain AII and BII in monomer II are numbered 52–72 and 73–102, respectively. (E) Close-up view of inter-monomer interactions in the representative model of hIns_2_. Hydrogen bonds are indicated by dashed black lines. (F) Intra-monomer hydrophobic interactions in monomers. The monomer I and II are indicated in cyan and green, respectively.
(DOCX)

Figure S2 **Determination of simulation parameters for MC/MD scheme.** (A) Superposition of the lowest energy configuration at 300 K (green) with that at other temperatures (blue). RMSDs (in nm) of backbone atoms are indicated in parentheses. (B) RMSFs (in nm) plotted for side chain atoms of hIns_2_ from 1 ns long MD simulations at various termperatures. (C) RMSDs (in nm) plotted for side chain atoms of hIns_2_ from MD simulations with various time lengths. The time lengths 1, 2 and 3 ns are shown as black, red and blue, respectively.
(DOCX)

Figure S3 **Protonation state space exploration.** (A) Flow chart of the protocol in the current work for determining the lowest energy protonation state. In general, the starting structure of protein complex for gas-phase calculations is generated from MD simulations in water (light blue background, steps 1–3). After selecting representative starting structures and a random generation of initial protonation states, structures for low energy gas phase protonation states are derived in an iterative procedure (blue background) beginning with high-temperature MD simulations in the gas phase. Subsequently, the lowest energy conformation within equally spaced time windows is obtained by geometry optimization. The optimized structures are then employed in the MC procedure using GB corrected force field energies and a Metropolis test to define the current lowest energy protonation state. For the next iteration, a new protonation state is generated. Convergence is reached when the program fails to generate a new protonation state for ten consecutive iterations. The procedure converges in a relatively small number of MC steps indicated by our current work on a protein complex and previous calculations of single similar-sized proteins [38]. (B) Probability that a pair of DFT conformers with ΔE_DFT_ less than 10 kJ/mol falls within ΔE_c_ in the GB corrected force field energies. The probability is calculated by counting the number of pairs falling within ΔE_c_. (C) The number of ionized residues (circles) in the most probable protonation states of the hIns_2_ as a function of the protein net charge (*q*). Standard deviation from the average is given as error bars. The minimum and the maximum numbers of possible ionized residues for each total charge are indicated by the green and the red lines, respectively. The vertical dashed blue line indicates the main charge state in ESI-MS [52].
(DOCX)

Figure S4 **0.075 ms long MD simulation in the gas phase of [hIns_2_]^6+^.** (A) Radius of gyration (*R*_g_) of the entire hIns_2_, of monomer I, and of monomer II. (B) Center-of-mass (COM) distance between monomers. (C) COM distances between monomers and β-sheet region. (D) RMSD (in nm) from the starting conformations of hIns_2_. (E) Number of hydrogen bonds within the complex. (F) Number of hydrogen bonds between monomers.
(DOCX)

Figure S5 **The largest essential motions of the protein complex along the combined water-and-gas-phase trajectories obtained from the 0.01 µs long simulation in water and the 0.075 ms long simulation in the gas phase (A), along the trajectory from the converged part (0.055 to 0.075 ms) of the simulation in the gas phase (B), and along the trajectory from the entire 0.075 ms long simulation in the gas phase (C).** The monomer I and II are presented as cyan and green trace models, respectively. The β-sheet regions are highlighted in orange. The fluctuations of the backbone atoms are depicted as red arrows.
(DOCX)

Figure S6 **Correlations between CCS and a variety of properties obtained from MD simulations in the gas phase of [hIns_2_]^6+^.** (A) Radius of gyration (*R*_g_) of the entire hIns_2_. (B) Solvent accessible surface area (SASA) of the entire hIns_2_. (C) The angle between the center of mass of monomer I – β-sheet region – monomer II. (D) Number of contact pairs between the carbon atoms of the monomers within 0.60 nm. (E) Number of hydrogen bonds within the complex. (F) Number of hydrogen bonds between monomers.
(DOCX)

Figure S7 **0.035 ms long independent MD simulations in the gas phase of [hIns_2_]^6+^.** (A) Models of [hIns_2_]^6+^ obtained from MD simulations in the gas phase (from left to right, at 0 µs, 5.31 µs, 20.52 µs and 34.2 µs). (B) Secondary structure analysis for [hIns_2_]^6+^. (C) The angle between the COM of monomer I – β-sheet region – monomer II. (D) CCS values. (E) Number of contact pairs between the carbon atoms of the monomers within 0.60 nm. The figure captions are same with the ones used in Figure 3.
(DOCX)

Figure S8 **0.035 ms long independent MD simulations in the gas phase of the [hIns_2_]^6+^.** (A) Models of [hIns_2_]^6+^ obtained from MD simulations in the gas phase (from left to right, at 0 µs, 7.68 µs, 19.68 µs, and 34.2 µs). (B) Secondary structure analysis for [hIns_2_]^6+^. (C) The angle between the COM of monomer I – β-sheet region – monomer II. (D) CCS values. (E) Number of contact pairs between the carbon atoms of the monomers within 0.60 nm. The figure captions are same with the ones used in Figure 3.
(DOCX)

Figure S9 **0.025 ms long independent MD simulations with GROMOS force field in the gas phase of [hIns_2_]^6+^.** (A) Models of [hIns_2_]^6+^ obtained from MD simulations in the gas phase (from left to right, at 0 µs, 7.89 µs, 19.6 µs, and 24.3 µs). (B) Secondary structure analysis for [hIns_2_]^6+^. (C) The angle between the COM of monomer I – β-sheet region – monomer II. (D) CCS values. (E) Number of contact pairs between the carbon atoms of the monomers within 0.60 nm. The figure captions are same with the ones used in Figure 3.
(DOCX)

Table S1 **The lowest energy protonation states for charge states from 1+ to 15+.** The positive and negative charged residues are indicated by “+” and “−”, respectively.
(DOCX)

Table S2 **Average structural properties of MD simulations in the gas phase of [hIns_2_]^6+^ with the lowest energy protonation state.** From left to right: length of simulation (Length in µs); radius of gyration (*R*_g_ in nm); radius of gyration of backbone atoms (*R*_g,BB_ in nm); radius of gyration of monomer I (*R*_g,MI_ in nm); radius of gyration of monomer II (*R*_g,MII_ in nm); collision cross section (CCS in nm^2^); total surface area (SA in nm^2^); center-of-mass distance between monomers (COM_P-P_ in nm); number of hydrogen bonds in protein-protein interface (HB_P-P_); number of hydrogen bonds in complex (HB); number of hydrogen bonds in complex (HB); number of contact pairs between the carbon atoms of the monomers defined by a cutoff of 0.60 nm (Cont_P-P_). Standard deviations were reported in parenthesis.
(DOC)

Table S3 **CCS values (in nm^2^) for various hIns_2_ structures and snapshots taken from the 0.075 ms long MD simulations in the gas phase.**
(DOC)

Table S4 **The number of all the possible protonation states for various charge states (q = 1+ to *q* = 15+) of hIns_2_ calculated by using the equation in ref. [53] are reported.**
(DOC)

Table S5 **GB corrected force field energy differences (ΔE_corr_) of the pairs of conformers whose DFT energy difference (ΔE_DFT_) is within 10 kJ/mol.**
(DOC)

Table S6 **Average structural properties of MD simulations in the gas phase of hIns_2_ at the main charge state with the most probable protonation states.** From left to right: radius of gyration (*R*_g_ in nm); radius of gyration of backbone atoms (*R*_g,BB_ in nm); radius of gyration of monomer I (*R*_g,MI_ in nm); radius of gyration of monomer II (*R*_g,MII_ in nm); collision cross section (CCS in nm^2^); total surface area (SA in nm^2^); center-of-mass distance between monomers (COM_P-P_ in nm); number of hydrogen bonds in protein-protein interface (HB_P-P_); number of hydrogen bonds in complex (HB); number of hydrogen bonds in complex (HB); number of contact pairs between the carbon atoms of the monomers defined by a cutoff of 0.60 nm (Cont_P-P_). Standard deviations are reported in parenthesis.
(DOC)

Text S1 **Supplemental methods.**
(DOC)
