# Supplementary material for: Molecular Simulation-Based Structural Prediction of Protein Complexes in Mass Spectrometry: The Human Insulin Dimer
Source: PLoS Comput Biol. 2014 Sep 11;10(9):e1003838. doi: 10.1371/journal.pcbi.1003838 (PMC4161290; doi:10.1371/journal.pcbi.1003838)
Supplement: Figure S1 — MD simulation of hIns2 in water. (A) Primary sequence of hIns2 (each monomer consists of two chains of 21 and 30 amino acids linked by 2 disulfide bridges derived from a precursor molecule). The letters colored in red and blue represent chargeable sites of acidic (E, D, and C-terminal) and basic groups (R, K, H, and N-terminal), respectively, in solution. (B) hIns2 X-ray structure (PDB ID: 1MSO [76]). Monomer I (residues 1–51) and II (residues 52–102) are colored in blue and red, respectively. Each insulin monomer is composed of two peptide chains (A and B, colored in dark and light, respectively) linked by two disulfide bonds (shown as green sticks, sulfur atom in yellow). (C) Backbone atoms RMSD (in nm) from the starting conformation of hIns2 during the 100 ns long MD simulation in water. RMSD of the entire hIns2, of monomer I, and of monomer II are colored in black, red, and green, respectively. (D) B-factor (in Å2) plotted for Cα atoms of hIns2 from MD simulation and X-ray. The last 5 ns long MD trajectory of hIns2 has been used in the calculation of B-factors. The experimental values are obtained from the X-ray structure data of hIns2 [76]. Residues of chain AI and BI in monomer I are numbered 1–21 and 22–51, respectively. Residues of chain AII and BII in monomer II are numbered 52–72 and 73–102, respectively. (E) Close-up view of inter-monomer interactions in the representative model of hIns2. Hydrogen bonds are indicated by dashed black lines. (F) Intra-monomer hydrophobic interactions in monomers. The monomer I and II are indicated in cyan and green, respectively. (DOCX) [file pcbi.1003838.s001.docx]

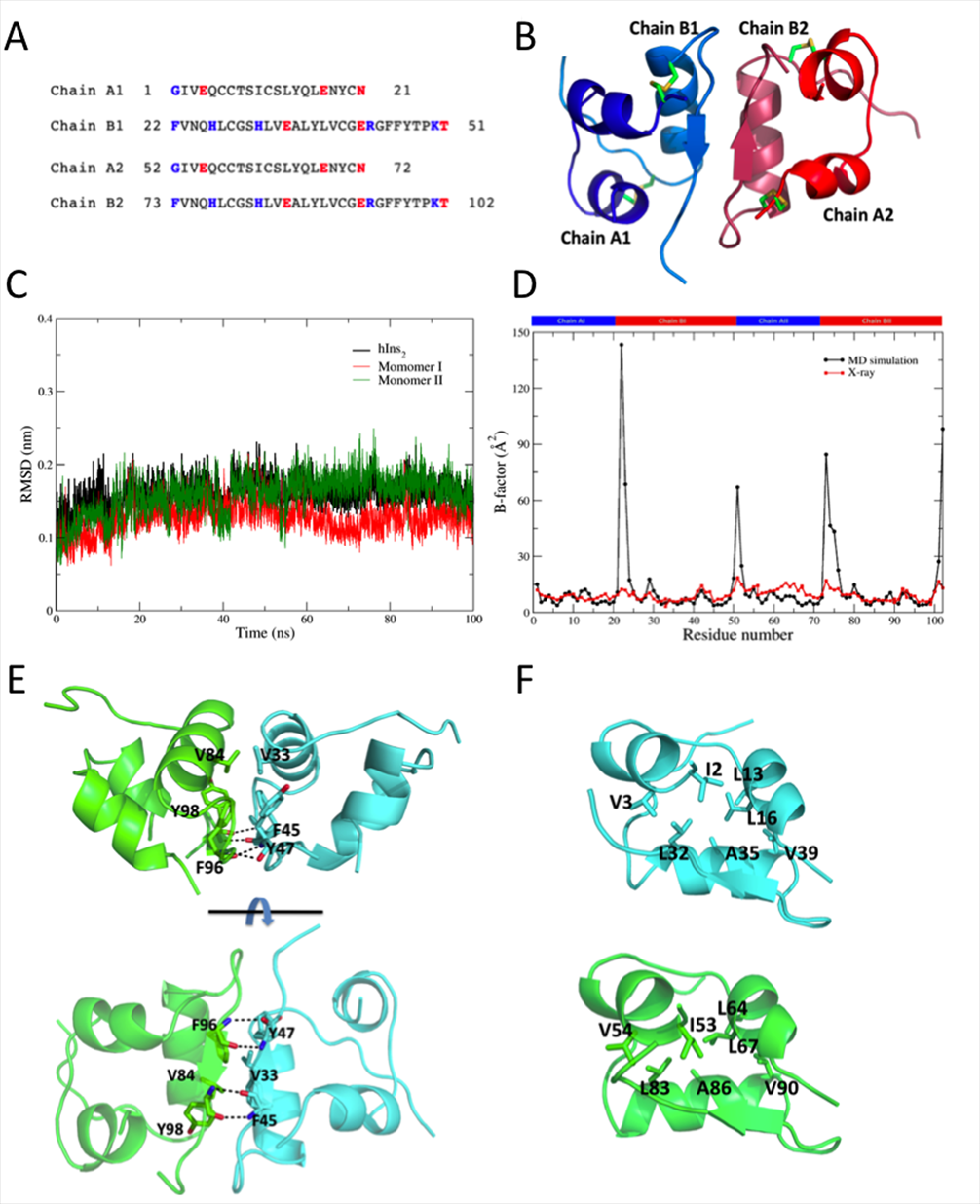


**Figure S1. MD simulation of hIns_2_ in water.** (A) Primary sequence of hIns_2_ (each monomer consists of two chains of 21 and 30 amino acids linked by 2 disulfide bridges derived from a precursor molecule). The letters colored in red and blue represent chargeable sites of acidic (E, D, and C-terminal) and basic groups (R, K, H, and N-terminal), respectively, in solution. (B) hIns_2_ X-ray structure (PDB ID: 1MSO [76]). Monomer I (residues 1-51) and II (residues 52-102) are colored in blue and red, respectively. Each insulin monomer is composed of two peptide chains (A and B, colored in dark and light, respectively) linked by two disulfide bonds (shown as green sticks, sulfur atom in yellow). (C) Backbone atoms RMSD (in nm) from the starting conformation of hIns_2_ during the 100 ns long MD simulation in water. RMSD of the entire hIns_2_, of monomer I, and of monomer II are colored in black, red, and green, respectively. (D) B-factor (in Å^2^) plotted for Cα atoms of hIns_2_ from MD simulation and X-ray. The last 5 ns long MD trajectory of hIns_2_ has been used in the calculation of B-factors. The experimental values are obtained from the X-ray structure data of hIns_2_ [76]. Residues of chain AI and BI in monomer I are numbered 1-21 and 22-51, respectively. Residues of chain AII and BII in monomer II are numbered 52-72 and 73-102, respectively. (E) Close-up view of inter-monomer interactions in the representative model of hIns_2_. Hydrogen bonds are indicated by dashed black lines. (F) Intra-monomer hydrophobic interactions in monomers. The monomer I and II are indicated in cyan and green, respectively.
