# Supplementary material for: Molecular Simulation-Based Structural Prediction of Protein Complexes in Mass Spectrometry: The Human Insulin Dimer
Source: PLoS Comput Biol. 2014 Sep 11;10(9):e1003838. doi: 10.1371/journal.pcbi.1003838 (PMC4161290; doi:10.1371/journal.pcbi.1003838)
Supplement: Figure S2 — Determination of simulation parameters for MC/MD scheme. (A) Superposition of the lowest energy configuration at 300 K (green) with that at other temperatures (blue). RMSDs (in nm) of backbone atoms are indicated in parentheses. (B) RMSFs (in nm) plotted for side chain atoms of hIns2 from 1 ns long MD simulations at various termperatures. (C) RMSDs (in nm) plotted for side chain atoms of hIns2 from MD simulations with various time lengths. The time lengths 1, 2 and 3 ns are shown as black, red and blue, respectively. (DOCX) [file pcbi.1003838.s002.docx]

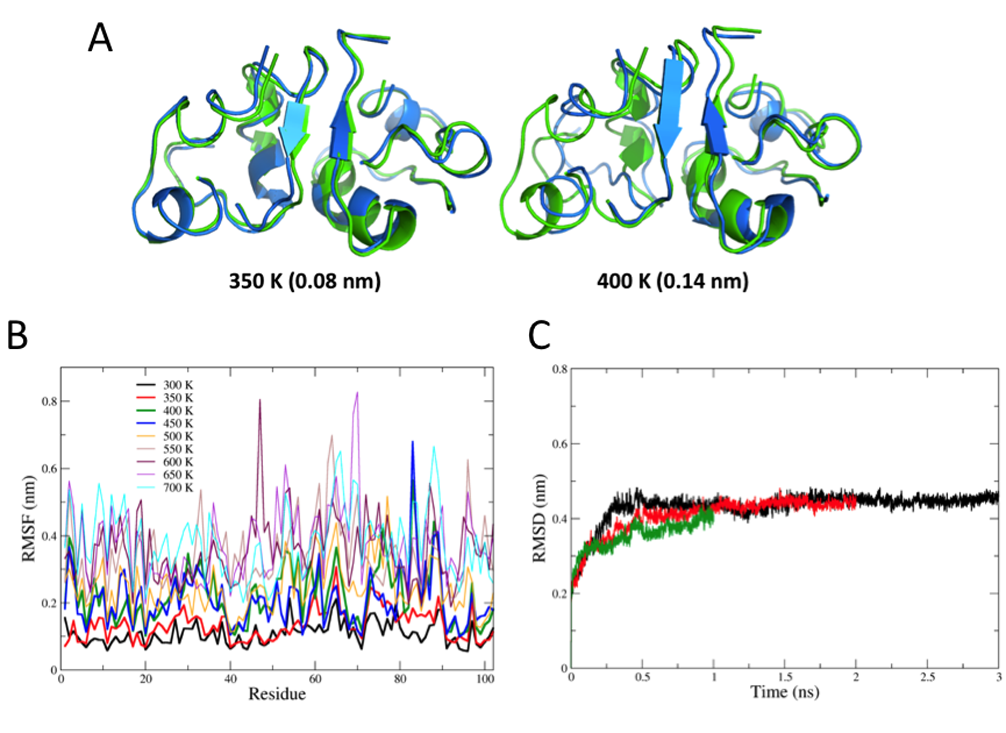


**Figure S2. Determination of simulation parameters for MC/MD scheme.** (A) Superposition of the lowest energy configuration at 300 K (green) with that at other temperatures (blue). RMSDs (in nm) of backbone atoms are indicated in parentheses. (B) RMSFs (in nm) plotted for side chain atoms of hIns_2_ from 1 ns long MD simulations at various termperatures. (C) RMSDs (in nm) plotted for side chain atoms of hIns_2_ from MD simulations with various time lengths. The time lengths 1, 2 and 3 ns are shown as black, red and blue, respectively.
