# Supplementary material for: Molecular Simulation-Based Structural Prediction of Protein Complexes in Mass Spectrometry: The Human Insulin Dimer
Source: PLoS Comput Biol. 2014 Sep 11;10(9):e1003838. doi: 10.1371/journal.pcbi.1003838 (PMC4161290; doi:10.1371/journal.pcbi.1003838)
Supplement: Figure S3 — Protonation state space exploration. (A) Flow chart of the protocol in the current work for determining the lowest energy protonation state. In general, the starting structure of protein complex for gas-phase calculations is generated from MD simulations in water (light blue background, steps 1–3). After selecting representative starting structures and a random generation of initial protonation states, structures for low energy gas phase protonation states are derived in an iterative procedure (blue background) beginning with high-temperature MD simulations in the gas phase. Subsequently, the lowest energy conformation within equally spaced time windows is obtained by geometry optimization. The optimized structures are then employed in the MC procedure using GB corrected force field energies and a Metropolis test to define the current lowest energy protonation state. For the next iteration, a new protonation state is generated. Convergence is reached when the program fails to generate a new protonation state for ten consecutive iterations. The procedure converges in a relatively small number of MC steps indicated by our current work on a protein complex and previous calculations of single similar-sized proteins [38]. (B) Probability that a pair of DFT conformers with ΔEDFT less than 10 kJ/mol falls within ΔEc in the GB corrected force field energies. The probability is calculated by counting the number of pairs falling within ΔEc. (C) The number of ionized residues (circles) in the most probable protonation states of the hIns2 as a function of the protein net charge (q). Standard deviation from the average is given as error bars. The minimum and the maximum numbers of possible ionized residues for each total charge are indicated by the green and the red lines, respectively. The vertical dashed blue line indicates the main charge state in ESI-MS [52]. (DOCX) [file pcbi.1003838.s003.docx]

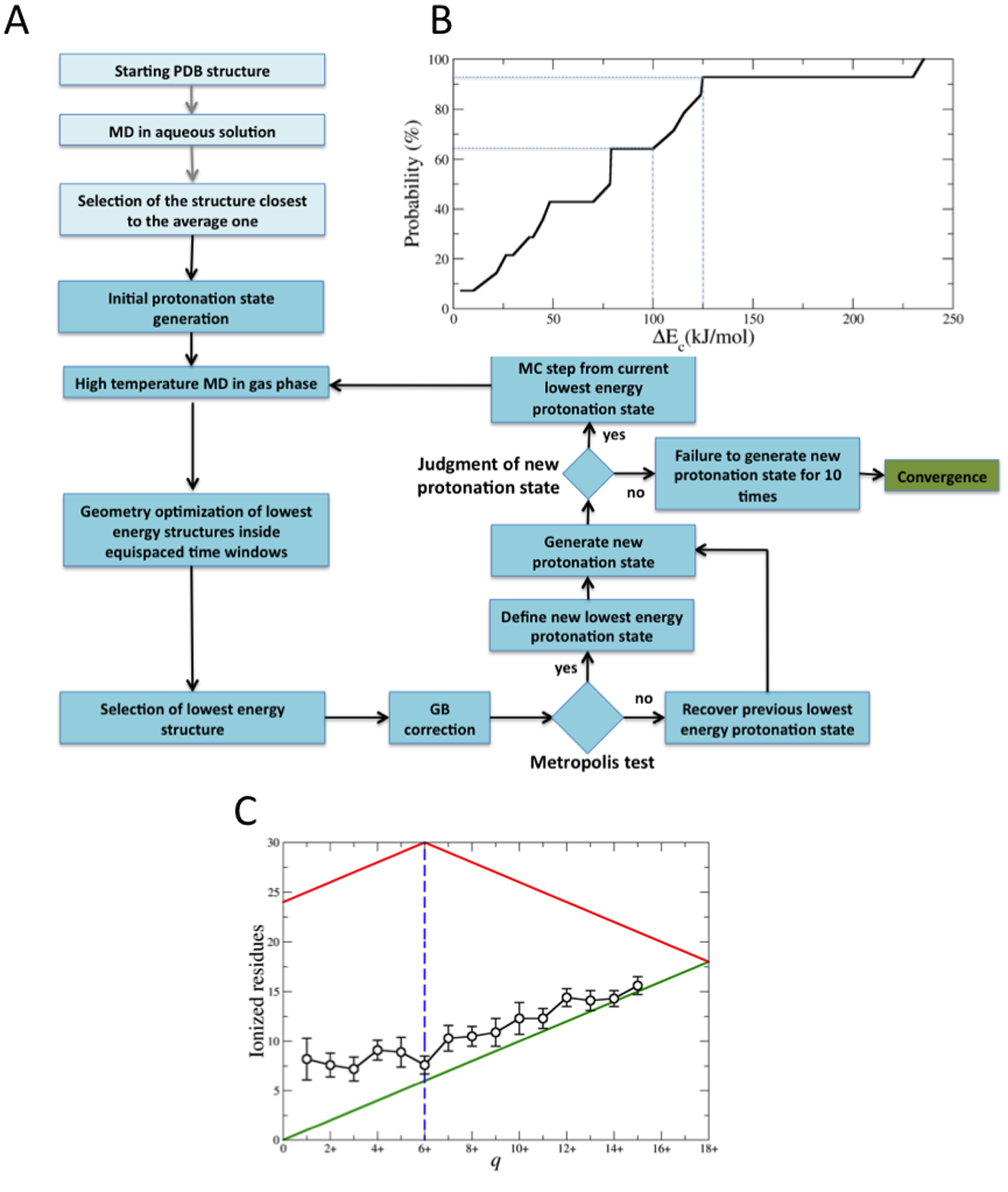


**Figure S3. Protonation state space exploration.** (A) Flow chart of the protocol in the current work for determining the lowest energy protonation state*.* In general, the starting structure of protein complex for gas-phase calculations is generated from MD simulations in water (light blue background, steps 1-3). After selecting representative starting structures and a random generation of initial protonation states, structures for low energy gas phase protonation states are derived in an iterative procedure (blue background) beginning with high-temperature MD simulations in the gas phase. Subsequently, the lowest energy conformation within equally spaced time windows is obtained by geometry optimization. The optimized structures are then employed in the MC procedure using GB corrected force field energies and a Metropolis test to define the current lowest energy protonation state. For the next iteration, a new protonation state is generated. Convergence is reached when the program fails to generate a new protonation state for ten consecutive iterations. The procedure converges in a relatively small number of MC steps indicated by our current work on a protein complex and previous calculations of single similar-sized proteins [38]. (B) Probability that a pair of DFT conformers with ΔE_DFT_ less than 10 kJ/mol falls within ΔE_c_ in the GB corrected force field energies. The probability is calculated by counting the number of pairs falling within ΔE_c_. (C) The number of ionized residues (circles) in the most probable protonation states of the hIns_2_ as a function of the protein net charge (*q*). Standard deviation from the average is given as error bars. The minimum and the maximum numbers of possible ionized residues for each total charge are indicated by the green and the red lines, respectively. The vertical dashed blue line indicates the main charge state in ESI-MS [52].
