# Supplementary material for: Molecular Simulation-Based Structural Prediction of Protein Complexes in Mass Spectrometry: The Human Insulin Dimer
Source: PLoS Comput Biol. 2014 Sep 11;10(9):e1003838. doi: 10.1371/journal.pcbi.1003838 (PMC4161290; doi:10.1371/journal.pcbi.1003838)
Supplement: Figure S4 — 0.075 ms long MD simulation in the gas phase of [hIns2]6+. (A) Radius of gyration (R g) of the entire hIns2, of monomer I, and of monomer II. (B) Center-of-mass (COM) distance between monomers. (C) COM distances between monomers and β-sheet region. (D) RMSD (in nm) from the starting conformations of hIns2. (E) Number of hydrogen bonds within the complex. (F) Number of hydrogen bonds between monomers. (DOCX) [file pcbi.1003838.s004.docx]

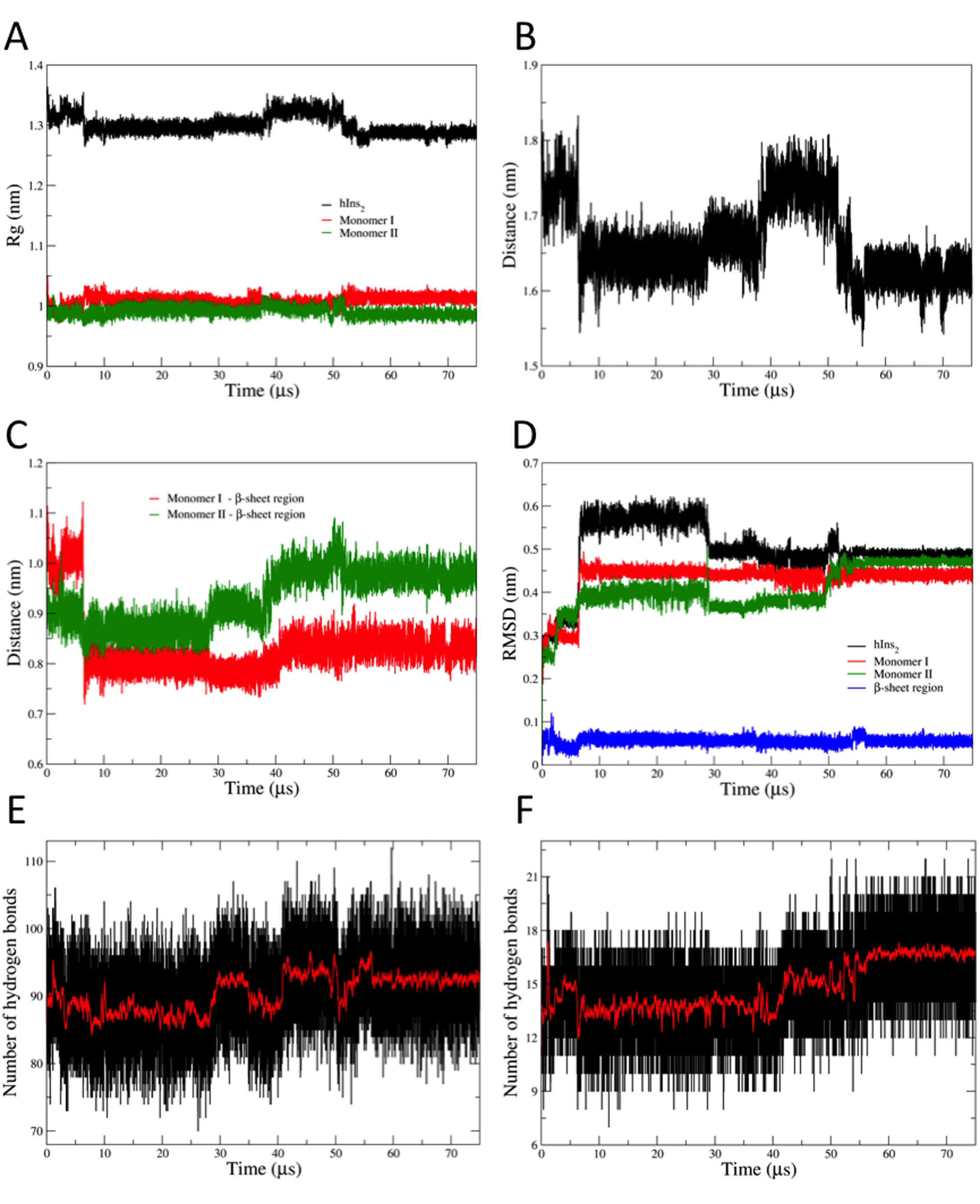


**Figure S4.** **0.075 ms long** **MD simulation in the gas phase of [hIns_2_]^6+^.** (A) Radius of gyration (*R*_g_) of the entire hIns_2_, of monomer I, and of monomer II. (B) Center-of-mass (COM) distance between monomers. (C) COM distances between monomers and β-sheet region. (D) RMSD (in nm) from the starting conformations of hIns_2_. (E) Number of hydrogen bonds within the complex. (F) Number of hydrogen bonds between monomers.
