# Supplementary material for: Molecular Simulation-Based Structural Prediction of Protein Complexes in Mass Spectrometry: The Human Insulin Dimer
Source: PLoS Comput Biol. 2014 Sep 11;10(9):e1003838. doi: 10.1371/journal.pcbi.1003838 (PMC4161290; doi:10.1371/journal.pcbi.1003838)
Supplement: Figure S5 — The largest essential motions of the protein complex along the combined water-and-gas-phase trajectories obtained from the 0.01 µs long simulation in water and the 0.075 ms long simulation in the gas phase (A), along the trajectory from the converged part (0.055 to 0.075 ms) of the simulation in the gas phase (B), and along the trajectory from the entire 0.075 ms long simulation in the gas phase (C). The monomer I and II are presented as cyan and green trace models, respectively. The β-sheet regions are highlighted in orange. The fluctuations of the backbone atoms are depicted as red arrows. (DOCX) [file pcbi.1003838.s005.docx]

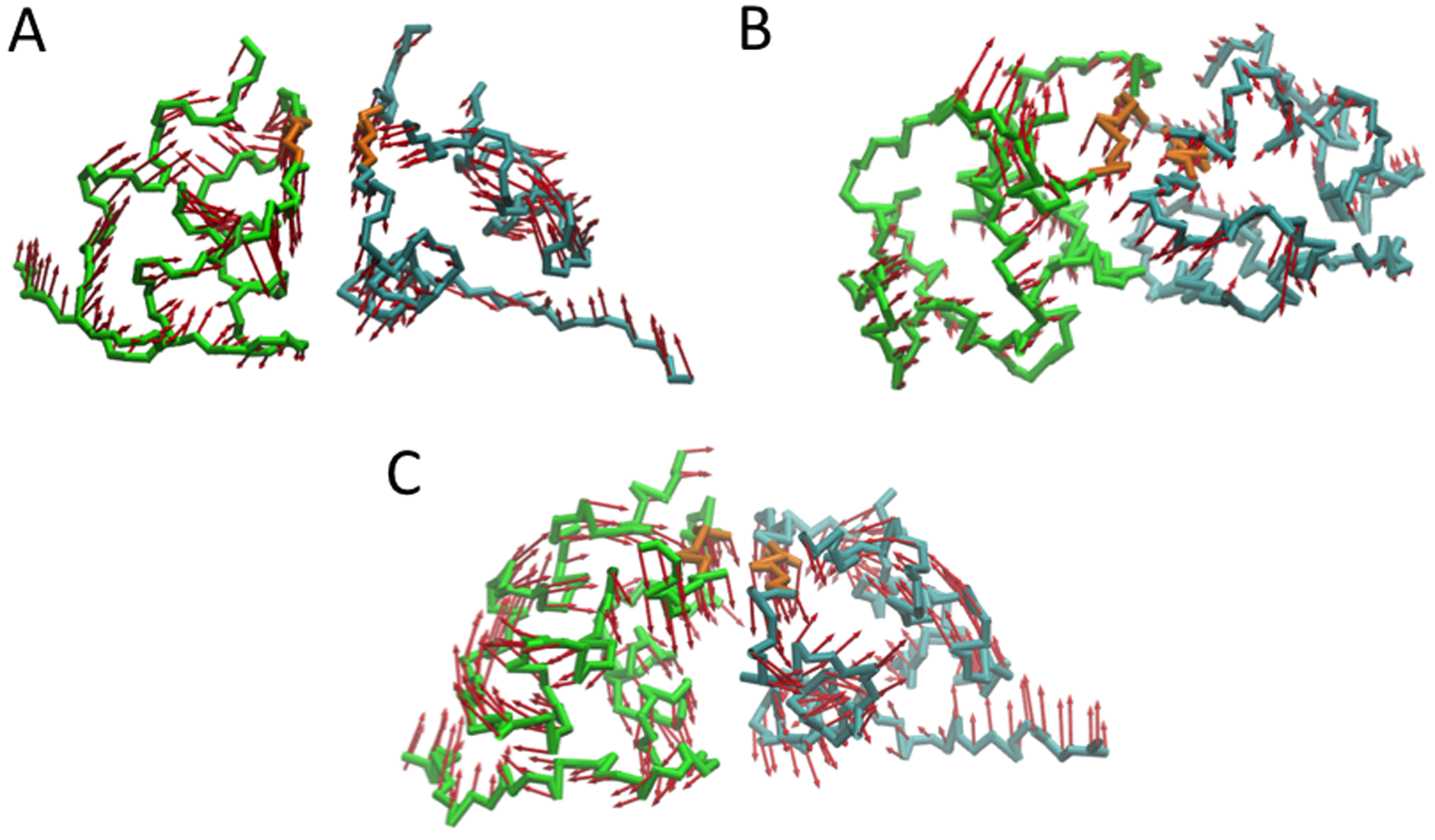


**Figure S5.** **The largest essential motions of the protein complex along the combined water-and-gas-phase trajectories obtained from the 0.01 μs long simulation in water and the 0.075 ms long simulation in the gas phase (A), along the trajectory from the converged part (0.055 to 0.075 ms) of the simulation in the gas phase (B), and along the trajectory from the entire 0.075 ms long simulation in the gas phase (C)**. The monomer I and II are presented as cyan and green trace models, respectively. The β-sheet regions are highlighted in orange. The fluctuations of the backbone atoms are depicted as red arrows.
