# Supplementary material for: Molecular Simulation-Based Structural Prediction of Protein Complexes in Mass Spectrometry: The Human Insulin Dimer
Source: PLoS Comput Biol. 2014 Sep 11;10(9):e1003838. doi: 10.1371/journal.pcbi.1003838 (PMC4161290; doi:10.1371/journal.pcbi.1003838)
Supplement: Figure S6 — Correlations between CCS and a variety of properties obtained from MD simulations in the gas phase of [hIns2]6+. (A) Radius of gyration (R g) of the entire hIns2. (B) Solvent accessible surface area (SASA) of the entire hIns2. (C) The angle between the center of mass of monomer I – β-sheet region – monomer II. (D) Number of contact pairs between the carbon atoms of the monomers within 0.60 nm. (E) Number of hydrogen bonds within the complex. (F) Number of hydrogen bonds between monomers. (DOCX) [file pcbi.1003838.s006.docx]

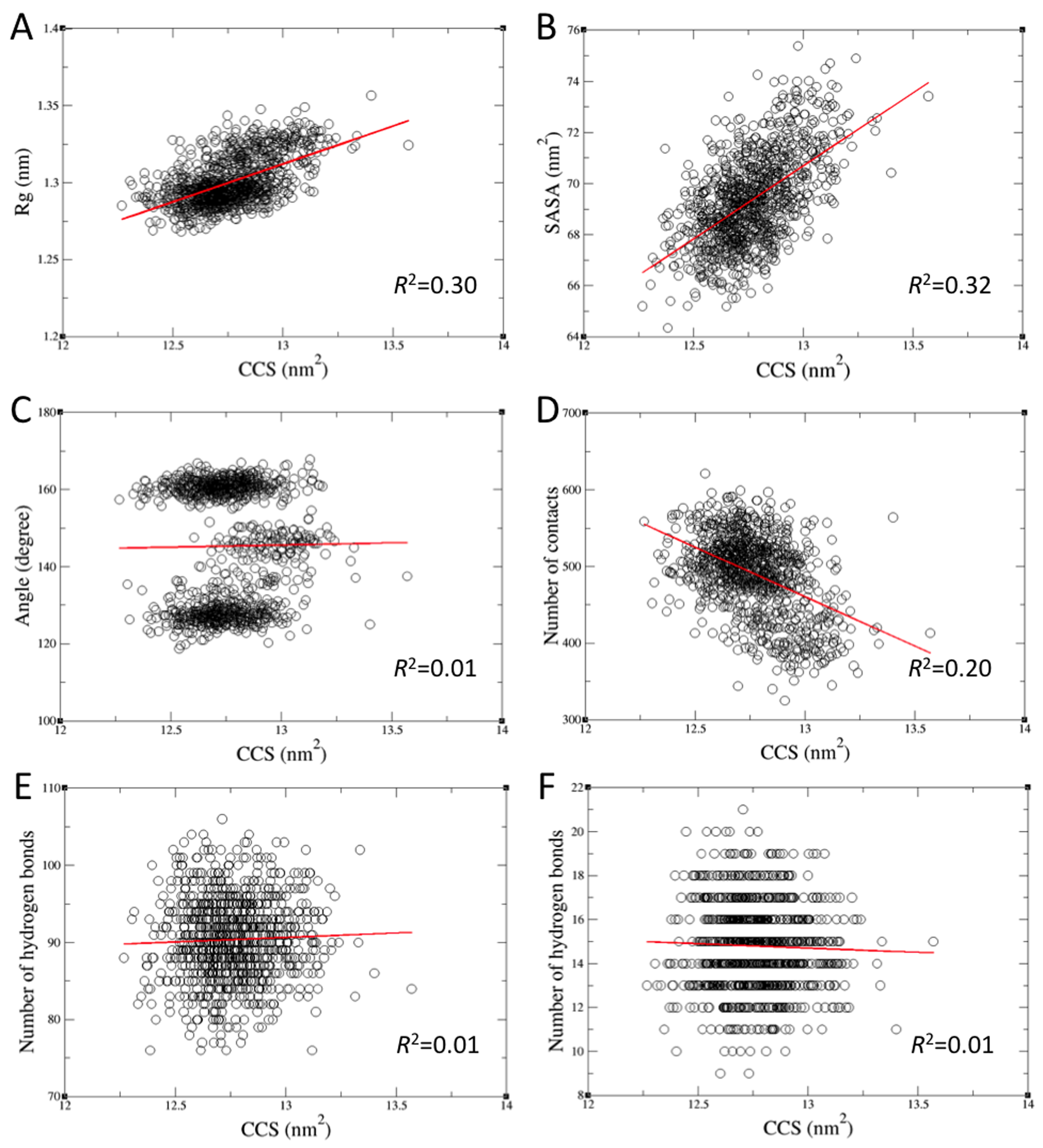


**Figure S6. Correlations between CCS and a variety of properties obtained from MD simulations in the gas phase of [hIns_2_]^6+^**. (A) Radius of gyration (*R*_g_) of the entire hIns_2_. (B) Solvent accessible surface area (SASA) of the entire hIns_2_. (C) The angle between the center of mass of monomer I – β-sheet region – monomer II. (D) Number of contact pairs between the carbon atoms of the monomers within 0.60 nm. (E) Number of hydrogen bonds within the complex. (F) Number of hydrogen bonds between monomers.
