# Supplementary material for: Molecular Simulation-Based Structural Prediction of Protein Complexes in Mass Spectrometry: The Human Insulin Dimer
Source: PLoS Comput Biol. 2014 Sep 11;10(9):e1003838. doi: 10.1371/journal.pcbi.1003838 (PMC4161290; doi:10.1371/journal.pcbi.1003838)
Supplement: Figure S8 — 0.035 ms long independent MD simulations in the gas phase of the [hIns2]6+. (A) Models of [hIns2]6+ obtained from MD simulations in the gas phase (from left to right, at 0 µs, 7.68 µs, 19.68 µs, and 34.2 µs). (B) Secondary structure analysis for [hIns2]6+. (C) The angle between the COM of monomer I – β-sheet region – monomer II. (D) CCS values. (E) Number of contact pairs between the carbon atoms of the monomers within 0.60 nm. The figure captions are same with the ones used in Figure 3. (DOCX) [file pcbi.1003838.s008.docx]

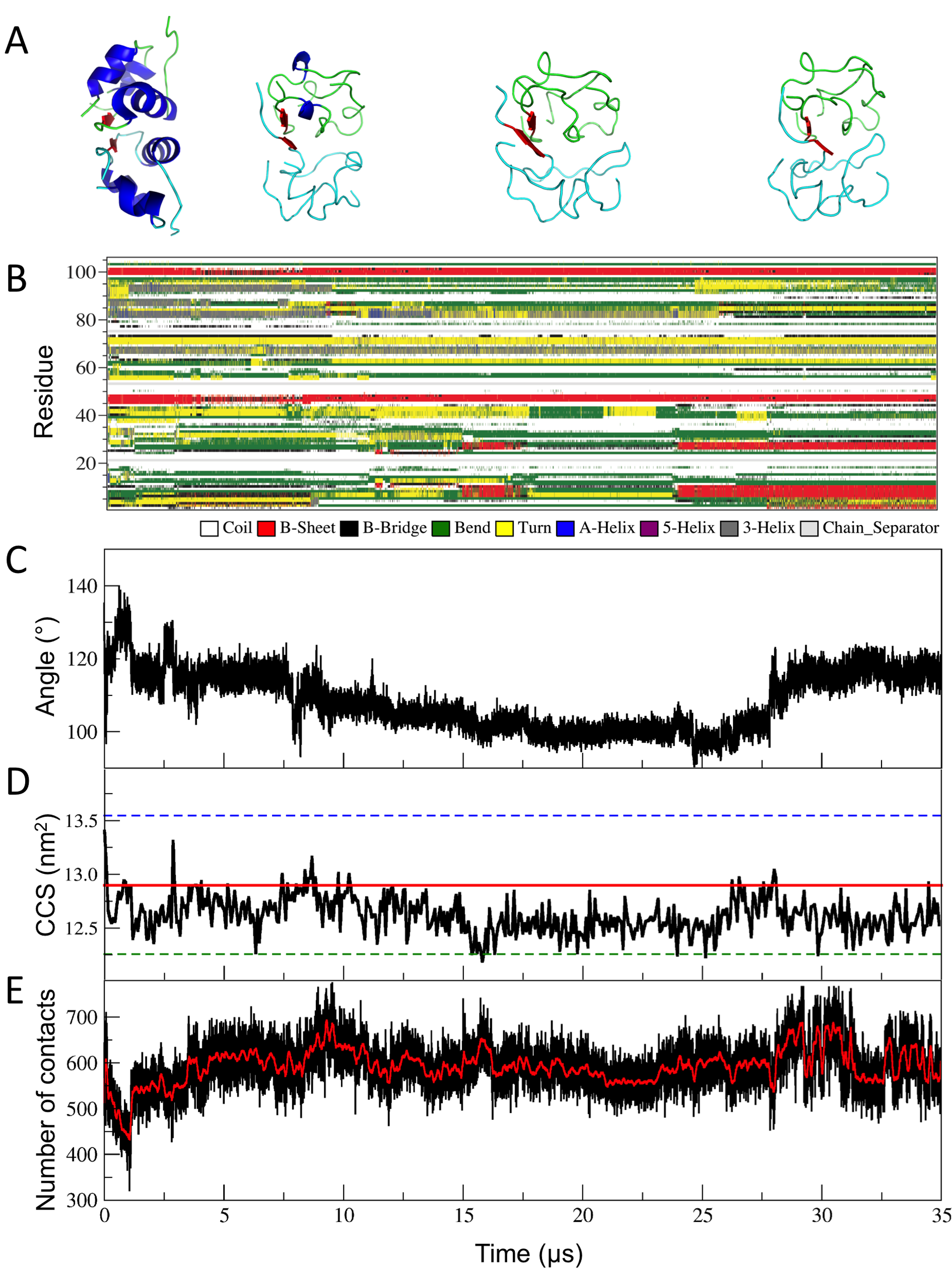


**Figure S8. 0.035 ms long independent MD simulations in the gas phase of the [hIns_2_]^6+^*.*** (A) Models of [hIns_2_]^6+^ obtained from MD simulations in the gas phase (from left to right, at 0 μs, 7.68 μs, 19.68 μs, and 34.2 μs). (B) Secondary structure analysis for [hIns_2_]^6+^. (C) The angle between the COM of monomer I – β-sheet region – monomer II. (D) CCS values. (E) Number of contact pairs between the carbon atoms of the monomers within 0.60 nm. The figure captions are same with the ones used in Figure 3.
