# Supplementary material for: Molecular Simulation-Based Structural Prediction of Protein Complexes in Mass Spectrometry: The Human Insulin Dimer
Source: PLoS Comput Biol. 2014 Sep 11;10(9):e1003838. doi: 10.1371/journal.pcbi.1003838 (PMC4161290; doi:10.1371/journal.pcbi.1003838)
Supplement: Table S1 — The lowest energy protonation states for charge states from 1+ to 15+. The positive and negative charged residues are indicated by “+” and “−”, respectively. (DOCX) [file pcbi.1003838.s010.docx]

**Table S1.** The lowest energy protonation states for charge states from 1+ to 15+. The positive and negative charged residues are indicated by “+” and “-”, respectively.

|  | **1+** | **2+** | **3+** | **4+** | **5+** | **6+** | **7+** | **8+** | **9+** | **10+** | **11+** | **12+** | **13+** | **14+** | **15+** |
| --- | --- | --- | --- | --- | --- | --- | --- | --- | --- | --- | --- | --- | --- | --- | --- |
| Number of charged amino acids | 11 | 8 | 7 | 10 | 7 | 8 | 9 | 10 | 11 | 14 | 13 | 14 | 13 | 14 | 15 |
| N-terminal G1 | + | + | + | + |  | + |  | + | + | + | + | + | + | + | + |
| E4 | - |  |  |  |  |  |  |  |  |  | - |  |  |  |  |
| Q5 |  |  |  |  |  |  |  |  |  |  |  | + | + | + | + |
| Q15 |  |  |  |  |  |  |  |  |  | + |  |  |  | + | + |
| E17 |  |  |  |  |  |  |  |  |  |  |  |  |  |  |  |
| C-terminal N21 | - | - | - | - | - | - | - |  |  |  |  |  |  |  |  |
| N-terminal F22 | + |  |  | + | + | + | + | + | + | + | + | + | + | + | + |
| Q25 |  |  |  |  |  |  |  |  |  |  |  | + |  |  |  |
| H26 |  |  |  |  |  |  | + | + | + |  | + | + | + | + | + |
| H31 |  |  |  |  |  |  |  |  | + | + | + | + | + | + | + |
| E34 |  | - |  |  |  |  |  |  |  |  |  |  |  |  |  |
| E42 |  |  |  |  |  |  |  |  |  |  |  |  |  |  |  |
| R43 |  |  |  |  |  |  | + | + |  | + | + |  | + | + |  |
| K50 | + | + | + | + | + | + |  | + | + | + | + | + | + | + | + |
| C-terminal T51 | - |  |  |  |  |  |  |  |  | - |  | - |  |  |  |
| N-terminal G52 | + |  | + | + | + | + | + | + | + | + | + | + | + | + | + |
| E55 |  |  | - | - |  |  |  |  |  |  |  |  |  |  |  |
| Q56 |  |  |  |  |  |  |  |  |  |  |  | + |  |  | + |
| Q66 |  |  |  |  |  |  |  |  |  |  |  |  |  |  | + |
| E68 |  |  |  |  |  |  |  |  |  |  |  |  |  |  |  |
| C-terminal N72 | - |  |  |  |  |  |  |  | - | - |  |  |  |  |  |
| N-terminal F73 | + |  | + | + | + | + |  | + | + | + | + | + | + | + | + |
| Q76 |  | + |  |  |  |  |  |  |  |  |  |  |  | + |  |
| H77 |  |  |  | + | + |  | + | + | + | + | + | + | + | + | + |
| H82 |  | + |  |  |  | + | + |  |  | + | + |  | + |  | + |
| E85 |  | - |  |  |  |  |  |  |  |  |  |  |  |  |  |
| E93 |  |  |  |  |  |  |  |  |  |  |  |  |  |  |  |
| R94 |  |  |  |  |  |  | + |  | + | + | + | + | + | + | + |
| K101 | + | + | + | + | + | + | + | + | + | + | + | + | + | + | + |
| C-terminal T102 | - |  |  | - |  |  |  | - |  |  |  |  |  |  |  |
