# Supplementary material for: Molecular Simulation-Based Structural Prediction of Protein Complexes in Mass Spectrometry: The Human Insulin Dimer
Source: PLoS Comput Biol. 2014 Sep 11;10(9):e1003838. doi: 10.1371/journal.pcbi.1003838 (PMC4161290; doi:10.1371/journal.pcbi.1003838)
Supplement: Table S2 — Average structural properties of MD simulations in the gas phase of [hIns2]6+ with the lowest energy protonation state. From left to right: length of simulation (Length in µs); radius of gyration (R g in nm); radius of gyration of backbone atoms (R g,BB in nm); radius of gyration of monomer I (R g,MI in nm); radius of gyration of monomer II (R g,MII in nm); collision cross section (CCS in nm2); total surface area (SA in nm2); center-of-mass distance between monomers (COMP-P in nm); number of hydrogen bonds in protein-protein interface (HBP-P); number of hydrogen bonds in complex (HB); number of hydrogen bonds in complex (HB); number of contact pairs between the carbon atoms of the monomers defined by a cutoff of 0.60 nm (ContP-P). Standard deviations were reported in parenthesis. (DOC) [file pcbi.1003838.s011.doc]

**Table S2.** Average structural properties of MD simulations in the gas phase of [hIns_2_]^6+^ with the lowest energy protonation state. From left to right: length of simulation (Length in μs); radius of gyration (*R*_g_ in nm); radius of gyration of backbone atoms (*R*_g,BB_ in nm); radius of gyration of monomer I (*R*_g,MI_ in nm); radius of gyration of monomer II (*R*_g,MII_ in nm); collision cross section (CCS in nm^2^); total surface area (SA in nm^2^); center-of-mass distance between monomers (COM_P-P_ in nm); number of hydrogen bonds in protein-protein interface (HB_P-P_); number of hydrogen bonds in complex (HB); number of hydrogen bonds in complex (HB); number of contact pairs between the carbon atoms of the monomers defined by a cutoff of 0.60 nm (Cont_P-P_). Standard deviations were reported in parenthesis.

|  | **Length** | ***R*_g_** | ***R*_g,BB_** | ***R*_g,MI_** | ***R*_g,MII_** | **CCS** | **SA** | **COM_P-P_** | **HB_P-P_** | **HB** | **Cont_P-P_** |
| --- | --- | --- | --- | --- | --- | --- | --- | --- | --- | --- | --- |
| **[hIns_2_]^6+^** | 75 | 1.30(0.01) | 1.25(0.01) | 1.01(0.01) | 0.99(0.01) | 12.8(0.2) | 69.24(1.76) | 1.66(0.02) | 14.8(1.9) | 90.7(5.0) | 492.9(50.5) |
|  | 35 | 1.31(0.01) | 1.26(0.01) | 1.02(0.01) | 1.01(0.01) | 12.9(0.2) | 69.53(1.28) | 1.64(0.02) | 13.7(2.1) | 91.5(5.9) | 529.2(55.0) |
|  | 35 | 1.27(0.01) | 1.23(0.01) | 1.02(0.01) | 0.98(0.01) | 12.6(0.1) | 68.01(1.16) | 1.55(0.02) | 13.4(1.8) | 90.3(5.0) | 587.5(44.7) |
| **[hIns_2_]_Gro_^6+^,*^a^*** | 25 | 1.20(0.01) | 1.16(0.01) | 0.97(0.01) | 1.01(0.01) | 11.7(0.3) | 64.54(1.91) | 1.35(0.03) | 17.3(3.1) | 89.3(5.8) | 604.9(75.2) |
| **hIns_2,wat_*^b^*** | 0.1 | 1.37(0.01) | 1.32(0.01) | 1.03(0.01) | 1.03(0.01) | 16.9(0.1) | 76.84 (1.95) | 1.82(0.03) | 5.2(1.4) | 64.2(4.1) | 510.1(10.1) |

*^a^* Structural properties of hIns_2_ obtained from gas-phase MD simulations using GROMOS force field.

*^b^* Structural properties of hIns_2_ obtained from MD simulations in water.
