# Supplementary material for: Molecular Simulation-Based Structural Prediction of Protein Complexes in Mass Spectrometry: The Human Insulin Dimer
Source: PLoS Comput Biol. 2014 Sep 11;10(9):e1003838. doi: 10.1371/journal.pcbi.1003838 (PMC4161290; doi:10.1371/journal.pcbi.1003838)
Supplement: Table S3 — CCS values (in nm2) for various hIns2 structures and snapshots taken from the 0.075 ms long MD simulations in the gas phase. (DOC) [file pcbi.1003838.s012.doc]

**Table S3.** CCS values (in nm^2^) for various hIns_2_ structures and snapshots taken from the 0.075 ms long MD simulations in the gas phase.

|  | CCS |
| --- | --- |
| X-ray*^a^* | 16.6 |
| Rep_water*^b^* | 16.9 |
| Exp*^c^* | 12.9 |
| 0 μs | 13.4 |
| 0.9 μs | 12.7 |
| 5.7 μs | 12.6 |
| 8.1 μs | 12.7 |
| 27.6 μs | 12.6 |
| 31.2 μs | 12.4 |
| 36.3 μs | 12.6 |
| 42.6 μs | 13.0 |
| 54.9 μs | 12.9 |
| 75.0 μs | 12.8 |

*^a^* The hIns_2_ X-ray structure at 1.0 Å (PDB ID: 1MSO [76]).

*^b^* The representative structure from our MD simulation in solvent.

*^c^* The experimental CCS value from ref. [52].
