# Supplementary material for: Molecular Simulation-Based Structural Prediction of Protein Complexes in Mass Spectrometry: The Human Insulin Dimer
Source: PLoS Comput Biol. 2014 Sep 11;10(9):e1003838. doi: 10.1371/journal.pcbi.1003838 (PMC4161290; doi:10.1371/journal.pcbi.1003838)
Supplement: Table S4 — The number of all the possible protonation states for various charge states (q = 1+ to q = 15+) of hIns2 calculated by using the equation in ref. [53] are reported. (DOC) [file pcbi.1003838.s013.doc]

**Table S4.** The number of all the possible protonation states for various charge states (q=1+ to *q*=15+) of hIns_2_ calculated by using the equation in ref. [53] are reported.

| ***q*** | **Number of protonation states** |
| --- | --- |
| 15+ | 4,060 |
| 14+ | 22,950 |
| 13+ | 132,084 |
| 12+ | 578,595 |
| 11+ | 2,018,376 |
| 10+ | 5,838,174 |
| 9+ | 14,298,020 |
| 8+ | 30,040,989 |
| 7+ | 54,626,100 |
| 6+ | 86,493,008 |
| 5+ | 119,759,832 |
| 4+ | 145,422,675 |
| 3+ | 155,117,520 |
| 2+ | 145,422,675 |
| 1+ | 119,759,850 |
