# Supplementary material for: Molecular Simulation-Based Structural Prediction of Protein Complexes in Mass Spectrometry: The Human Insulin Dimer
Source: PLoS Comput Biol. 2014 Sep 11;10(9):e1003838. doi: 10.1371/journal.pcbi.1003838 (PMC4161290; doi:10.1371/journal.pcbi.1003838)
Supplement: Table S5 — GB corrected force field energy differences (ΔEcorr) of the pairs of conformers whose DFT energy difference (ΔEDFT) is within 10 kJ/mol. (DOC) [file pcbi.1003838.s014.doc]

**Table S5.** GB corrected force field energy differences (ΔE_corr_) of the pairs of conformers whose DFT energy difference (ΔE_DFT_) is within 10 kJ/mol.

| **Index** | **ΔE_corr_ (kJ/mol)** |
| --- | --- |
| 1 | -124.9 |
| 2 | -48.4 |
| 3 | -78.5 |
| 4 | -3.9 |
| 5 | -79.0 |
| 6 | -115.4 |
| 7 | -21.6 |
| 8 | -44.9 |
| 9 | -123.9 |
| 10 | -79.0 |
| 11 | -26.4 |
| 12 | -110.2 |
| 13 | -38.1 |
| 14 | -235.3 |
