# Supplementary material for: Molecular Simulation-Based Structural Prediction of Protein Complexes in Mass Spectrometry: The Human Insulin Dimer
Source: PLoS Comput Biol. 2014 Sep 11;10(9):e1003838. doi: 10.1371/journal.pcbi.1003838 (PMC4161290; doi:10.1371/journal.pcbi.1003838)
Supplement: Table S6 — Average structural properties of MD simulations in the gas phase of hIns2 at the main charge state with the most probable protonation states. From left to right: radius of gyration (R g in nm); radius of gyration of backbone atoms (R g,BB in nm); radius of gyration of monomer I (R g,MI in nm); radius of gyration of monomer II (R g,MII in nm); collision cross section (CCS in nm2); total surface area (SA in nm2); center-of-mass distance between monomers (COMP-P in nm); number of hydrogen bonds in protein-protein interface (HBP-P); number of hydrogen bonds in complex (HB); number of hydrogen bonds in complex (HB); number of contact pairs between the carbon atoms of the monomers defined by a cutoff of 0.60 nm (ContP-P). Standard deviations are reported in parenthesis. (DOC) [file pcbi.1003838.s015.doc]

**Table S6.** Average structural properties of MD simulations in the gas phase of hIns_2_ at the main charge state with the most probable protonation states. From left to right: radius of gyration (*R*_g_ in nm); radius of gyration of backbone atoms (*R*_g,BB_ in nm); radius of gyration of monomer I (*R*_g,MI_ in nm); radius of gyration of monomer II (*R*_g,MII_ in nm); collision cross section (CCS in nm^2^); total surface area (SA in nm^2^); center-of-mass distance between monomers (COM_P-P_ in nm); number of hydrogen bonds in protein-protein interface (HB_P-P_); number of hydrogen bonds in complex (HB); number of hydrogen bonds in complex (HB); number of contact pairs between the carbon atoms of the monomers defined by a cutoff of 0.60 nm (Cont_P-P_). Standard deviations are reported in parenthesis.

|  | ***R*_g_** | ***R*_g,BB_** | ***R*_g,MI_** | ***R*_g,MII_** | **CCS** | **SA** | **COM_P-P_** | **HB_P-P_** | **HB** | **Cont_P-P_** |
| --- | --- | --- | --- | --- | --- | --- | --- | --- | --- | --- |
| **Prot 1** | 1.30(0.01) | 1.25(0.01) | 1.01(0.01) | 0.99(0.01) | 12.8(0.2) | 69.24(1.76) | 1.66(0.02) | 14.8(1.9) | 90.7(5.0) | 492.9(50.5) |
| **Prot 2** | 1.33(0.01) | 1.29(0.01) | 1.05(0.01) | 1.01(0.01) | 13.3(0.2) | 70.25(1.37) | 1.73(0.02) | 12.5(1.6) | 91.9(4.6) | 535.0(34.6) |
| **Prot 3** | 1.29(0.01) | 1.25(0.01) | 1.00(0.01) | 1.02(0.01) | 13.0(0.1) | 70.23(1.13) | 1.61(0.03) | 14.6(1.8) | 91.8(4.4) | 561.1(22.7) |
| **Prot 4** | 1.25(0.01) | 1.20(0.01) | 1.05(0.01) | 1.00(0.01) | 12.6(0.2) | 68.22(1.68) | 1.34(0.03) | 13.1(1.9) | 96.0(5.2) | 587.4(40.3) |
| **Prot 5** | 1.25(0.01) | 1.20(0.01) | 1.02(0.01) | 1.00(0.01) | 12.7(0.1) | 67.41(1.33) | 1.42(0.01) | 16.2(1.7) | 85.6(4.0) | 661.9(25.3) |
| **hIns_2,wat_*^a^*** | 1.37(0.01) | 1.32(0.01) | 1.03(0.01) | 1.03(0.01) | 16.9(0.1) | 76.84 (1.95) | 1.82(0.03) | 5.21(1.4) | 64.2(4.1) | 510.1(10.1) |

*^a^* Structural properties of hIns_2_ obtained from MD simulation in water.
