# Supplementary material for: Molecular Simulation-Based Structural Prediction of Protein Complexes in Mass Spectrometry: The Human Insulin Dimer
Source: PLoS Comput Biol. 2014 Sep 11;10(9):e1003838. doi: 10.1371/journal.pcbi.1003838 (PMC4161290; doi:10.1371/journal.pcbi.1003838)
Supplement: Text S1 — Supplemental methods. (DOC) [file pcbi.1003838.s016.doc]

**Text S1. Supplemental methods**

**1. MD simulations of hIns_2_ in water**

The structure of hIns_2_ appeared to be equilibrated already after ~10 ns as indicated by plots of the backbone heavy atoms root mean square deviations (RMSD) as a function of simulated time (Figure S1C). The structure whose backbone is the closest to that of the average conformation (RMSD=0.6 Å) was extracted from the trajectory at a time between 10 and 100 ns to be used as the starting structure for gas-phase MD simulations. The B-factor and monomer-monomer interaction analyses of the complex in water were presented in Figure S1D and in Figures S1E and S1F, respectively. Averages of structural properties were reported in Table S2.

**2. Determination of simulation parameters for MC/MD scheme**

We optimized the parameters (temperature and sampling time length) in the hybrid Monte Carlo/Molecular Dynamics (MC/MD) scheme for the exploration of the protonation state space (see next section for a more detailed description). To this end, 1 ns long MD simulations in gas phase were carried out on hIns_2_ for a randomly generated protonation state with temperatures of 300, 350 and 400 K. As the internal temperature of ions just emitted from droplets is associated with uncertainties, there is, up to now, no clear connection between simulation and experimental temperatures . The RMSD plot of side chain atoms starts to fluctuate around an average value of 0.38±0.02 nm after 0.5 ns. Similar behavior was observed for longer simulations, including 2 and 3 ns (Figure S2), suggesting that the economical 1 ns long simulation has similar performance in covering a variety of conformations for a given protonation state. The structures for all tested temperatures are well conserved (RMSD<0.2 nm, Figure S2). A calculation of the root mean square fluctuations (RMSF) of side chain atoms (Figure S2B) as in ref. and , leads us to the observation that, as expected, increasing the temperature increases the fluctuations of side chains.

**3. Protonation state space exploration**

A flow chart describing the protocol for protonation state space exploration is reported in Figure S3A. The initial gas phase charge distribution is randomly generated by following the protocol used in ref. . For each protonation state considered, a 1 ns long MD simulation at 400 K was performed to allow for side chain reorganization. The resulting trajectory is split into 60 equally spaced time windows. In each window the geometry of the lowest energy conformation is optimized using conjugate gradient geometry optimization. The energy of the conformer with the lowest value among the 60 optimized structures is recalculated with the gas-phase basicity (GB) corrected force field and compared with that of the previous lowest energy (reference) protonation state (for the first loop the structure is always accepted). If the energy of the current protonation state is lower than the one of the reference protonation state, the current protonation state would be the new reference, i.e. the “lowest energy” protonation state, otherwise the protonation state would be rejected. To start the new loop, an MC step is performed on the current reference protonation state. The MC step consists of the deprotonation of a randomly selected protonated ionizable residue (neutralization of an ionized basic site or ionization of a neutral acidic site) and the protonation of another randomly selected protonated ionizable residue (ionization of a neutral basic site or the neutralization of an ionized acidic site), resulting in a proton shift between two residues. This procedure maintains the fixed total charge. The iterative procedure stops when no new protonation states are generated in ten consecutive iterations. For each value of net charge, this procedure converges in a relatively small number of MC steps (from 1000 to 3000). Our MC/MD calculations on single proteins and peptides indicate that the GB corrected force field allows discriminating between high and low energy protonation states. However, this scheme does not allow measuring small energy differences and, thus, identifying the single lowest energy protonation state . To cope with this problem, we carried out an analysis of the protonation state properties within a given cutoff (see following section for the determination of the cutoff), which yields with high confidence to include the lowest energy protonation state. We refer to these protonation states as the “most probable” protonation states. The analysis and discussion of protonation state properties presented in this work are based on a 125 kJ/mol cutoff (see following section). Only few structures are within this cutoff (about 5 to 10 out of thousands).

Zwitterionic states of the most probable protonation states are mostly retained, especially for low charge states (1+ to 5+, Figure S3C). However, they are totally abolished in high charge states (13+ to 15+, Figure S3C). Comparison of the lowest energy protonation states indicates that some of the positive sites are very conserved across the majority of the charge states, i.e. the N-terminal residues (G1, F22, G52 and F73), K50 and K101 (Table S2).

**4. Determination of the energy cutoff for the identification of the most probable protonation states**

The data dispersion of the correlation between DFT energies and GB corrected force field energies (Figure 1B) indicates that the corrected force field allows to discriminate between high and low energy protonation states but not to appreciate small energy differences. To study the dispersion and obtain a reasonable cutoff criterion for the selection of the most probable protonation states, we adopted the following procedure. First, we identified the conformers (14 pairs out of 60 conformers) whose DFT energy differences (ΔE_DFT_) are within 10 kJ/mol. Then, the corresponding GB corrected force field energy difference (ΔE_corr_) in each pair of conformers was calculated (Table S5). We performed a statistical analysis of these force field energy differences in order to generate the probability distribution of conformers falling into different energy cutoffs (ΔE_c_). As shown in Figure S3B, most of the pairs of conformers (92.9 %) with ΔE_DFT_ less than 10 kJ/mol fall within the ΔE_c_ of 125 kJ/mol.

Notice that the standard error (σ) of our linear regression between DFT and corrected force field (Figure 1B) is 63.7 kJ/mol. Thus, if we assume a normal distribution of the DFT energies around the estimation obtained from the corrected force field, there is a confidence of 68.2%, 95.4%, and 99.8% that the DFT energy is within σ, 2σ (127.4 kJ/mol) and 3σ (191.1 kJ/mol) from the estimation, respectively. The value of 2σ is very close to the ΔE_c_ (125 kJ/mol) derived from our protocol. Thus the discussion of protonation state properties presented in this work is based on a cutoff of 125 kJ/mol.

**5. Calculation of the *GB_app_***

The *GB_app,i_* of the *i-*th residue in a protein with total charge *q* is defined as

,

where *GB_i_* is the GB of the *i*-th amino acid in the gas phase and is the energy of the protein with that residue protonated (non-protonated). In contrast to the original formulation developed for a coarse-grained representation of an unfolded protein , we included in the calculation of the *GB_app_* all the classical energy terms included in a force field (FF). Vibrational corrections were not taken into account. The justification for this choice has been discussed previously in the literature .

**6. MD simulations in the gas phase of [hIns_2_]^6+^ with the most probable protonation states**

To check the sampling and convergence of the gas-phase MD trajectories, we calculated the cosine contents of the first two eigenvectors . These account for 63% of the backbone motions in the 0.075 ms long MD simulations. The cosine contents of the eigenvectors are 0.036 and 0.003 for the 0.075 ms long simulations. The values for two additional independent 0.035 ms long simulations and the 0.025 ms long simulation with GROMOS 43a1 force field are 0.127 and 0.197, 0.128 and 0.110, and 0.231 and 0.268, respectively.

In order to compare dynamic properties of different protonation states with the same charge state, MD simulations on five alternative lower energy protonation states (the most probable protonation states identified with a 125 kJ/mol cutoff) at the main charge state (q=6+), with charges located on different residues, have been carried out (Prot1 (the lowest energy protonation state) to Prot5, see Table S6).

**Supplemental References**

1. Arcella A, Portella G, Ruiz ML, Eritja R, Vilaseca M, et al. (2012) Structure of triplex DNA in the gas phase. J Am Chem Soc 134: 6596-6606.

2. Seeliger D, Haas J, de Groot BL (2007) Geometry-based sampling of conformational transitions in proteins. Structure 15: 1482-1492.

3. Smith GR, Sternberg MJ, Bates PA (2005) The relationship between the flexibility of proteins and their conformational states on forming protein-protein complexes with an application to protein-protein docking. J Mol Biol 347: 1077-1101.

4. Marchese R, Grandori R, Carloni P, Raugei S (2012) A computational model for protein ionization by electrospray based on gas-phase basicity. J Am Soc Mass Spectrom 23: 1903-1910.

5. Marchese R, Grandori R, Carloni P, Raugei S (2010) On the Zwitterionic Nature of Gas-Phase Peptides and Protein Ions. PLoS Comput Biol 6: 1-11.

6. Schnier PD, Gross DS, Williams ER (1995) On the maximum charge state and proton transfer reactivity of peptide and protein ions formed by electrospray ionization. J Am Soc Mass Spectrom 6: 1086-1097.

7. Berka K, Laskowski R, Riley KE, Hobza P, Vondrasek J (2009) Representative Amino Acid Side Chain Interactions in Proteins. A Comparison of Highly Accurate Correlated ab Initio Quantum Chemical and Empirical Potential Procedures. J Chem Theory Comput 5: 982-992.

8. Hess B (2002) Convergence of sampling in protein simulations. Physical Review E 65: 031910-031920.

9. Scott WRP, Hunenberger PH, Tironi IG, Mark AE, Billeter SR, et al. (1999) The GROMOS biomolecular simulation program package. Journal of Physical Chemistry A 103: 3596-3607.
